# Pluronic F127 Micelles Co-encapsulating a Brachydin-Enriched Fraction and Curcumin: A Self-assembled Platform for Light-assisted Antileishmanial Activity

**DOI:** 10.1021/acsomega.6c05278

**Published:** 2026-10-01

**Authors:** Ingrid Maria Ribeiro de Lima, Aline Santana Figueredo, Lucilene Amorim Silva, Luciana Magalhães Rebelo Alencar, Cláudia Quintino da Rocha, Renato Sonchini Gonçalves

**Affiliations:** † Laboratory of Chemistry of Natural Products, Department of Chemistry, Federal University of Maranhão, São Luís 65080-805, Brazil; ‡ Laboratory of Immunophysiology, Center for Biological and Health Sciences, Federal University of Maranhão, São Luís 65080-805, Brazil; § Laboratory of Biophysics and Nanosystems, Department of Physics, Federal University of Maranhão, São Luís 65080-805, Brazil; ∥ Department of Engineering and Exact Sciences, Palotina Sector, Federal University of Paraná (UFPR), Palotina 85950-000, Parana, Brazil

## Abstract

This study developed an associative Pluronic F127-based formulation co-loaded with a brachydin-enriched fraction and curcumin (FBC) for preliminary light-assisted antileishmanial evaluation. The formulation showed high apparent active-material recovery after centrifugation and filtration, as estimated by HPLC-PDA. DLS analysis indicated a polydisperse colloidal profile, with an apparent mean hydrodynamic diameter of approximately 406.5 nm and a number-weighted smaller population centered at approximately 12.1 nm. FTIR data were consistent with formulation-related changes in the vibrational environment of the hydrophobic active materials, while AFM and SEM provided complementary morphological information on deposited or dried samples. Together, these data support an interpretative F127-based self-assembly model, without establishing a fully uniform colloidal population or a definitively resolved internal micellar architecture. In vitro cytotoxicity assays using GM07492A human lung fibroblasts indicated low basal cytotoxicity under dark conditions, whereas light exposure produced concentration-dependent viability reductions at higher formulation concentrations. Antileishmanial assays against *Leishmania amazonensis* promastigotes revealed a marked light-associated response, mainly in formulations containing the brachydin-enriched fraction. FB showed the highest potency under light exposure, with a formulation-based IC_50_ of 18.63 μg mL^–1^, whereas FBC showed an IC_50_ decrease from 72.74 to 34.33 μg mL^–1^. Thus, although FBC was not more potent than FB, it combined light-associated antileishmanial activity, high apparent active-material recovery, and favorable macroscopic formulation behavior. Because FB and FBC contain a brachydin-enriched fraction rather than purified individual brachydins, the biological responses should be interpreted as preliminary formulation-level effects. Further studies involving direct reactive oxygen species measurements, intracellular amastigote assays, formal selectivity analysis, fraction standardization, and long-term colloidal stability evaluation are still required before therapeutic efficacy can be inferred.

## Graphical abstract

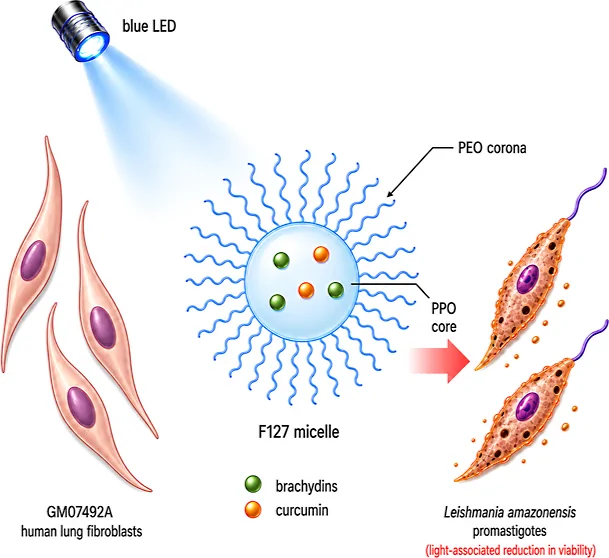

## 1 Introduction

Leishmaniasis is a neglected tropical disease caused by protozoan parasites of the genus *Leishmania*, affecting millions of people worldwide, particularly in low- and middle-income regions.^1,2^ The disease presents in different clinical forms, ranging from cutaneous lesions to severe mucocutaneous and visceral manifestations, often leading to significant morbidity and social stigma.^3^ Despite decades of research, current therapeutic options remain limited and are associated with high toxicity, prolonged treatment regimens, and the continuous emergence of drug-resistant strains.^4,5^

In recent years, natural products have emerged as promising sources of bioactive compounds with antiparasitic potential. Among them, flavonoids have attracted considerable attention due to their diverse biological activities, including anti-inflammatory, antioxidant, and antileishmanial effects.^6,7^ In particular, dimeric flavonoids such as brachydins, isolated from *Arrabidaea brachypoda* (currently accepted as *Fridericia platyphylla*), have demonstrated significant activity against *Leishmania* species, inducing structural and metabolic alterations that lead to parasite death.^8,9^ However, their therapeutic application is heavily limited by poor aqueous solubility and low bioavailability.

Curcumin, a polyphenolic compound derived from *Curcuma longa*, has also been extensively investigated for its broad pharmacological profile, including antiparasitic activity.^10^ Its mechanism of action against *Leishmania* involves the induction of oxidative stress, mitochondrial dysfunction, and modulation of cellular signaling pathways.^11^ Notably, curcumin exhibits prominent photosensitizing properties, making it a highly promising candidate for photodynamic therapy (PDT), a strategy based on the generation of reactive oxygen species (ROS) upon light activation.^12,13^ Nevertheless, similar to brachydins, curcumin suffers from physicochemical limitations that restrict its clinical application.

Photodynamic therapy has emerged as an alternative approach for the treatment of infectious diseases, including leishmaniasis, due to its ability to induce localized oxidative damage with minimal systemic toxicity.^14^ Recent advances have highlighted the potential of nanocarrier-assisted PDT systems to enhance the stability, delivery, and photodynamic efficiency of photosensitizers, significantly improving therapeutic outcomes in infectious and parasitic diseases.^15,16^ Specifically, studies have demonstrated the efficacy of nanoparticulate curcumin coupled with PDT against species such as *Leishmania* major.^17^ However, the development of efficient, multifaceted PDT systems for *Leishmania* remains challenging, particularly regarding the simultaneous cellular delivery of highly hydrophobic active compounds.

In this context, nanostructured drug delivery systems, especially polymeric micelles, have been widely explored to overcome the poor aqueous solubility, chemical instability, and limited bioavailability of hydrophobic bioactive compounds. Among these systems, PEO–PPO–PEO triblock copolymers, commonly known as poloxamers or pluronics, occupy an important position because their amphiphilic architecture enables temperature—and concentration-dependent self-association in aqueous media. In these assemblies, the relatively hydrophobic poly­(propylene oxide) (PPO) segments contribute to the formation of drug-associating domains, whereas the hydrated poly­(ethylene oxide) (PEO) chains provide steric stabilization and improve dispersion in aqueous environments.^18–20^

Pluronic F127 has been extensively investigated as a pharmaceutical and nanotechnological excipient for poorly water-soluble compounds because of its high PEO content, biocompatibility, and ability to form micellar or gel-like systems depending on concentration and temperature. Previous studies have shown that F127-based systems can improve the apparent solubility, colloidal dispersion, and delivery performance of hydrophobic molecules, including natural products and photosensitizer-like compounds. In particular, curcumin-loaded Pluronic systems have been reported to enhance curcumin dispersion in aqueous media and to modulate its aggregation behavior, illustrating the usefulness of PEO–PPO-based carriers for polyphenolic compounds with limited water solubility.^21–23^

Within this broader context, the novelty of the present study does not reside in the use of Pluronic F127 itself, which is already well established as a micellar carrier, but rather in the associative co-encapsulation of brachydin-enriched fraction and curcumin in a single F127-based formulation intended for light-assisted antileishmanial evaluation. This approach seeks to integrate two hydrophobic natural products with distinct biological and physicochemical features into a simple self-assembled platform, while recognizing that the resulting colloidal organization must be interpreted in relation to the extensive prior literature on PEO–PPO nanocarriers and their concentration—and temperature-dependent behavior.^18,20,23^

Despite the individual potential of brachydins and curcumin, the novelty of the present work should be interpreted within the broader context of previously reported F127-based and curcumin-based systems. The formulation of hydrophobic natural products in Pluronic F127 micelles is already well established, and previous work from our group demonstrated that F127 can encapsulate brachydin-rich fractions and improve their antileishmanial applicability.^24^ Thus, the use of F127 itself should not be considered the main novelty of this study.

In parallel, curcumin has been investigated as a photosensitizer or light-responsive bioactive compound in photodynamic protocols, including nanocarrier-assisted approaches against *Leishmania* species and broader antimicrobial PDT applications.^25,26^ These previous studies support the relevance of curcumin for light-assisted antiparasitic strategies, but they do not directly correspond to the present F127-based co-loaded formulation containing both brachydins and curcumin.

Based on this context, the specific contribution of the present study is the associative co-encapsulation of brachydins and curcumin in a single Pluronic F127-based formulation and the preliminary evaluation of its light-associated antileishmanial activity against *Leishmania amazonensis* promastigotes. This contribution should be interpreted cautiously: FBC was not more potent than the brachydin-loaded formulation FB, and the present study did not directly demonstrate ROS generation, intracellular amastigote activity, or long-term nanoscale colloidal stability. Therefore, the current findings support formulation feasibility and preliminary light-associated biological activity, rather than a definitive selective and effective antiparasitic therapy.

## 2 Experimental Section

### 2.1 Materials

Pluronic F127 [poly­(ethylene oxide)–poly­(propylene oxide)–poly­(ethylene oxide), PEO–PPO–PEO triblock copolymer] was used as the polymeric carrier. Curcumin was obtained from commercial sources and used as received. Brachydins (dimeric flavonoids) were isolated from *F. platyphylla* roots as described below. Ethanol (analytical grade), methanol (HPLC grade), and formic acid were used for chromatographic and formulation procedures. Dichloromethane was employed during the extraction and fractionation process.

Ultrapure water was used for all aqueous preparations. Dulbecco’s modified Eagle medium (DMEM), fetal bovine serum (FBS), phosphate-buffered saline (PBS), trypsin, and antibiotic solution (penicillin–streptomycin and amphotericin B) were used for cell culture experiments. Schneider’s insect medium was used for *L. amazonensis* promastigote cultivation.

The MTT reagent [3-(4,5-dimethylthiazol-2-yl)-2,5-diphenyltetrazolium bromide] was used for cell viability and antiparasitic assays. Dimethyl sulfoxide (DMSO) was used for solubilization of formazan crystals and as a positive control in cytotoxicity assays. Pentamidine was used as a reference drug in antileishmanial assays. All reagents were of analytical grade and used without further purification unless otherwise stated.

### 2.2 Plant Sourcing

Roots of *F. platyphylla* were collected in April 2021 at Sant’Ana da Serra farm, located in João Pinheiro, Minas Gerais, Brazil.

Botanical identification was performed at the José Badine Herbarium, Federal University of Ouro Preto, by Dr. Maria Cristina Teixeira Braga Messias. A voucher specimen (no. 17,935) was deposited in the same herbarium. The collection and use of plant material were carried out in accordance with Brazilian regulations and registered in the National System for Managing Genetic Heritage and Associated Traditional Knowledge (SisGen, registration no. A451DE4).

### 2.3 Preparation of the Brachydin-Enriched Fraction

The extraction and fractionation procedures were performed as previously described.^8,24^ Briefly, the roots of *F. platyphylla* were dried and ground using a knife mill. The resulting powder was macerated in an ethanol/water mixture (7:3, v/v). After filtration, the ethanolic extract was collected and concentrated under reduced pressure at 40 °C to obtain the crude extract.

Subsequently, the crude extract was diluted in water and subjected to liquid–liquid partitioning with dichloromethane and methanol/water, yielding dichloromethane and hydroalcoholic fractions. The dichloromethane fraction, previously characterized as enriched in the dimeric flavonoids brachydin A, brachydin B, and brachydin C, was concentrated by rotary evaporation at 40 °C, resulting in a brown solid.^8,24^

In the present manuscript, the term “brachydins” is used operationally to refer to this brachydin-enriched dichloromethane fraction, rather than to a single purified compound or to individually isolated brachydin A, B, or C. The chromatographic profile and chemical characterization of this type of fraction were previously reported by our group.^24^ No additional HPLC-PDA chromatographic fingerprint, purity determination, or batch-to-batch reproducibility analysis was performed specifically for the present manuscript. Therefore, the biological responses reported here should be interpreted as formulation-level effects associated with the brachydin-enriched fraction incorporated into F127-based formulations, and not as effects attributable to an individual chemically pure brachydin.

### 2.4 Nanoencapsulation Method

The encapsulation of the brachydin-enriched fraction and curcumin into Pluronic F127 nanostructured micelles was carried out using the solid dispersion method, as illustrated in Figure 1A and previously reported for F127-based systems containing a brachydin-enriched fraction.^24^ Briefly, the F127 copolymer was dissolved in ethanol and maintained under constant stirring at 25 °C for 30 min.

**Figure 1:**
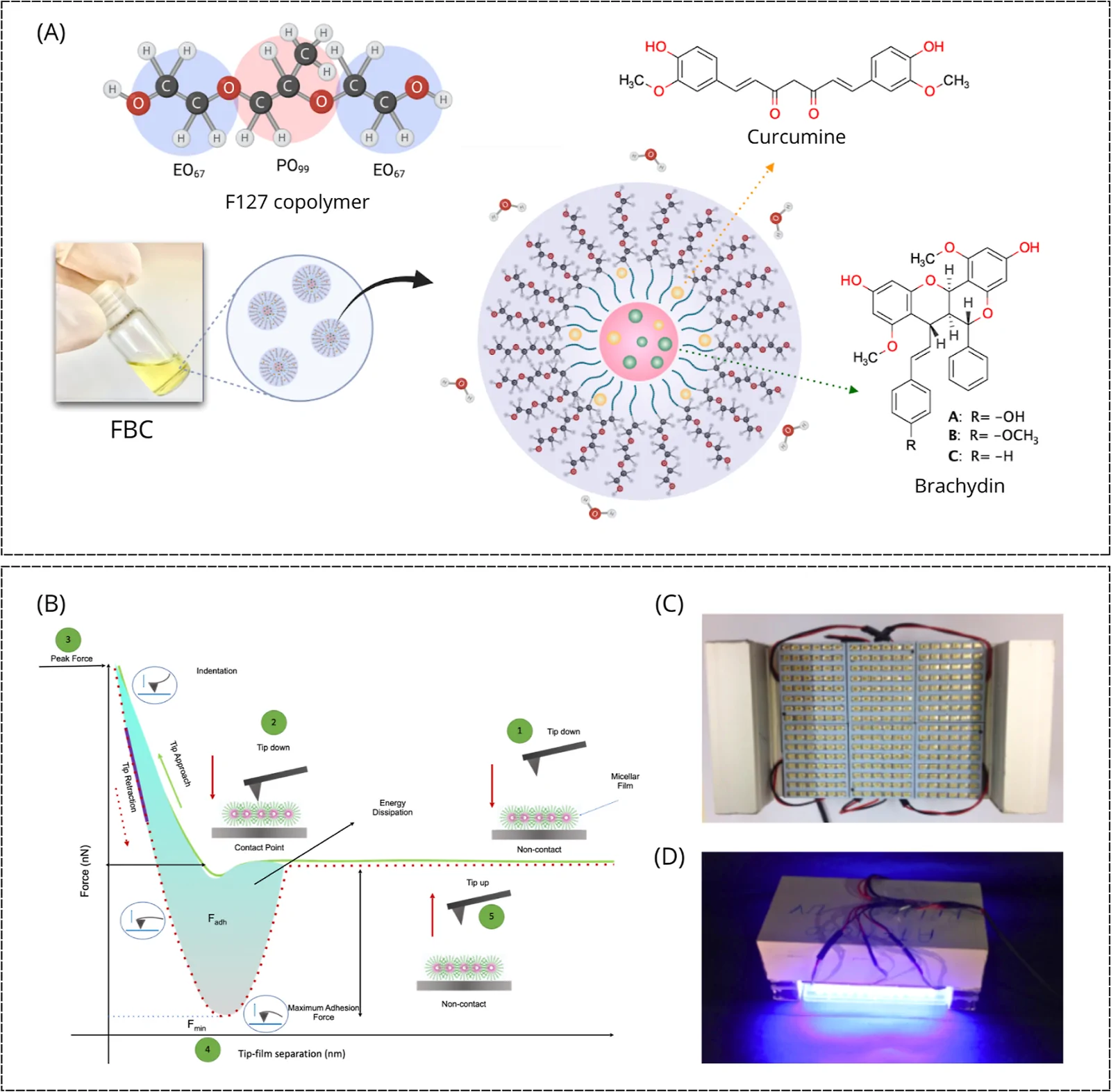
Overview of the F127-based co-loaded micellar system (FBC), AFM-based analysis, and light-assisted treatment setup. (A) Schematic illustration of FBC formation, in which the amphiphilic F127 copolymer (PEO–PPO–PEO) self-assembles into micellar assemblies with hydrophobic PPO-rich domains and a hydrated PEO corona, enabling the incorporation of the brachydin-enriched fraction and curcumin as hydrophobic active materials. Non-covalent interactions, including hydrophobic association and possible aromatic stacking, are represented as plausible contributors to active-material incorporation, but should not be interpreted as compound-specific interactions assigned to purified individual brachydins. The photograph shows the resulting liquid FBC formulation. (B) Schematic force–distance curve obtained by atomic force microscopy (AFM), highlighting the approach and retraction curves, energy dissipation, adhesion force (*F*_adh_), minimum force (*F*_min_), and contact region used for mechanical analysis. (C) Blue LED array system assembled for uniform light distribution. (D) Representative irradiation setup using a 96-well plate during light-assisted treatment. The photographs shown in panels A,C,D were taken by the authors during the present study and have not been previously published.

Four formulations were prepared and compared in the present study: empty F127 micelles (F), F127 micelles loaded with the brachydin-enriched fraction (FB), F127 micelles loaded with curcumin (FC), and F127 micelles co-loaded with the brachydin-enriched fraction and curcumin (FBC). In all cases, the final F127 concentration in the hydrated formulation was fixed at 0.5% (w/v), corresponding to 5.0 mg mL^–1^. For a standard 10 mL batch, this concentration corresponds to 50.0 mg of F127. When 20 mL batches were prepared, all component masses were proportionally doubled while maintaining the same final concentrations.

The FB formulation contained F127 at 5.0 mg mL^–1^ and the brachydin-enriched fraction at 60 μg mL^–1^, corresponding to 50.0 mg of F127 and 0.60 mg of the brachydin-enriched fraction in a 10 mL batch, with a fraction/F127 mass ratio of approximately 1:83.3. The FC formulation contained F127 at 5.0 mg mL^–1^ and curcumin at 37 μg mL^–1^, corresponding to 50.0 mg of F127 and 0.37 mg of curcumin in a 10 mL batch, with a curcumin/F127 mass ratio of approximately 1:135.1. The FBC formulation contained F127 at 5.0 mg mL^–1^, the brachydin-enriched fraction at 60 μg mL^–1^, and curcumin at 37 μg mL^–1^, corresponding to 50.0 mg of F127, 0.60 mg of the brachydin-enriched fraction, and 0.37 mg of curcumin in a 10 mL batch. For FBC, the total active material/F127 mass ratio, considering the brachydin-enriched fraction plus curcumin, was approximately 1:51.5.

For drug-loaded systems, ethanolic solutions of brachydins and/or curcumin were added to the polymer solution and stirred for an additional 30 min under the same conditions. The solvent was subsequently removed by rotary evaporation at 40 °C, yielding a thin polymeric film, which was further dried under vacuum for 24 h to ensure complete solvent removal. The resulting film was hydrated with ultrapure water and subjected to agitation in a Dubnoff shaking water bath (Thermo Scientific Precision DUB 15 L) at 50 cycles min^–1^ for 3 h. Based on the preliminary stability assay, hydration at 60 °C was selected for FBC preparation because it provided better macroscopic physical stability than hydration at room temperature. Empty F127 micelles were prepared under identical conditions, without the addition of active compounds, and used as vehicle controls in all experiments.

Unless otherwise specified, the concentrations reported for the cytotoxicity and antileishmanial assays refer to the nominal concentration of each formulation dispersion after dilution in the corresponding culture medium, and not to the isolated concentration of F127, brachydins, or curcumin alone. Therefore, comparisons among F, FB, FC, and FBC were performed using the same nominal formulation concentration range, while the equivalent concentrations of brachydins and/or curcumin were determined by the composition of each formulation described above. This clarification was included to avoid ambiguity when comparing single-loaded and co-loaded systems.

### 2.5 HPLC-PDA Analysis and Encapsulation Efficiency (EE %)

The quantification of the hydrophobic active materials in the liquid formulation, namely the brachydin-enriched fraction and curcumin, was performed by HPLC-PDA using a Shimadzu system (Shimadzu Corp., Kyoto, Japan) equipped with a binary pump and a UV–vis detector (SPD-10A). Chromatographic separation was achieved on a Luna C18 column (5 μm, 100 Å, 250 mm × 4.6 mm), using the chromatographic method previously reported for F127-based formulations containing a brachydin-enriched fraction.^24^

The mobile phase consisted of 2% formic acid in water and 2% formic acid in methanol, delivered at a flow rate of 1.0 mL min^–1^. The gradient elution program was as follows: 70–75% methanol from 0 to 10 min, 75–100% methanol from 10 to 20 min, followed by an isocratic step at 100% methanol for 10 min. The injection volume was 10 μL, and data acquisition and processing were performed using LC Solution software (Shimadzu).

Before HPLC-PDA analysis, formulation samples were centrifuged at 3000 rpm for 30 min to remove non-dispersed or precipitated material, followed by filtration through a 0.2 μm membrane filter. The filtrate was then analyzed by HPLC-PDA to quantify the active materials remaining in the liquid formulation after preparation. Thus, the non-incorporated fraction was operationally defined as the material removed during centrifugation and filtration prior to chromatographic analysis.

Encapsulation efficiency (EE %) was calculated by comparing the amount of active material detected in the centrifuged and filtered liquid formulation with the nominal amount initially added, according to eq 1:

1 $$EE\;\%=\left(\frac{m_{detected}}{m_{added}}\right)\times 100$$

where *m*_added_ represents the nominal mass of active material initially added to the formulation and *m*_detected_ represents the mass quantified by HPLC-PDA in the centrifuged and filtered formulation. For FBC, *m*_added_ corresponded to the total mass of the brachydin-enriched fraction plus curcumin initially added to the formulation. Because centrifugation and 0.2 μm filtration remove non-dispersed or precipitated material but do not fully distinguish molecularly free soluble compounds from micelle-associated compounds, the EE % reported here should be interpreted as an apparent formulation-level encapsulation/recovery parameter.

The chromatographic profile and chemical characterization of the brachydin-enriched fraction used as reference material were previously reported by our group.^24^ In the present manuscript, no additional full analytical validation, including new calibration curves, recovery studies, limits of detection, limits of quantification, or batch-to-batch reproducibility analysis, was performed specifically for the brachydin-enriched fraction. Therefore, the HPLC-PDA analysis was used to estimate the apparent recovery of the hydrophobic active materials in the final formulation, rather than to provide a new compound-specific purity or mass-balance validation for each individual brachydin.

### 2.6 Accelerated Stability Testing

To evaluate the influence of temperature on the physical and chemical stability of BC-loaded F127 micelles (FBC), accelerated stability testing was performed according to ANVISA Cosmetic Stability Guidelines and the United States Pharmacopeia.^27,28^ Briefly, 1.0 mL of each formulation was subjected to centrifugation at 3000 rpm (Centribio Co., Ltd., Shanghai, China) for 30 min at 25 ± 1 °C.

Samples were stored at room temperature (25 ± 3 °C) or under controlled refrigeration (5 ± 3 °C), with temperature monitored daily. Subsequently, the formulations were subjected to seven thermal cycles consisting of 24 h at 5 °C followed by 24 h at 25 °C.

Chemical stability was assessed by HPLC-PDA analysis, comparing BC concentrations at the end of the seventh cycle with those obtained on day 0 (EE % reference). Physical stability was evaluated based on homogeneity, absence of phase separation, and organoleptic characteristics, including appearance, color, and odor.

### 2.7 FTIR Analysis

Fourier-transform infrared (FTIR) spectroscopy was employed to evaluate possible changes in the vibrational environment of the active materials after formulation with F127. The spectra of the brachydin-enriched fraction/curcumin active mixture, empty F127 micelles (F), and FBC were recorded using an FTIR spectrophotometer (model Tracer-100, Shimadzu).

Samples were prepared in the solid state using the KBr pellet method. Spectra were acquired in the mid-infrared region (400–4000 cm^–1^), with a spectral resolution of 8 cm^–1^ and 50 scans per sample, at room temperature. The characteristic absorption bands of the active mixture and the polymeric matrix were analyzed to evaluate possible formulation-related interactions.^24^

### 2.8 Particle Size and ζ Potential

The average hydrodynamic diameter (*D*_h_), polydispersity index (PDI), and ζ potential of the F127-based formulations were determined by dynamic light scattering (DLS) using a Litesizer 500 instrument (Anton Paar GmbH, Graz, Austria; Module BM 10), equipped with a 40 mW semiconductor laser (λ = 658 nm). Measurements were carried out in aqueous medium at 25 °C.

DLS analyses were performed using the formulations as prepared, without prior dilution, in order to avoid concentration-dependent disruption, reorganization, or dilution-induced changes in Pluronic F127 micellar assemblies. The final F127 concentration during particle size and ζ potential measurements was 0.5% (w/v), corresponding to the polymer concentration used in the liquid formulation. For FBC, the formulation contained the brachydin-enriched fraction and curcumin at final concentrations of 60 and 37 μg mL^–1^, respectively. Empty F127 micelles (F) and the drug-loaded formulations were analyzed under the same preparation and measurement conditions.

Particle-size measurements were performed using disposable measurement cells. The dispersant was defined as water at 25 °C, with a refractive index of 1.330267, viscosity of 0.000890 Pa s, and relative permittivity of 78.368. The material setting was kept as unknown material, without defined refractive index or absorption coefficient. The instrument was configured in automatic measurement-angle mode, and the processed measurements used side-scatter geometry. The target temperature was 25 °C, with no additional equilibration time before acquisition.

The analysis model was set as general, and PDI values were obtained from cumulants analysis using the advanced cumulant model. The apparent mean hydrodynamic diameter and PDI were obtained from cumulants analysis, whereas the size-distribution profile shown for FBC was interpreted from the number-weighted distribution generated in the instrument report. Measurements were acquired using up to 15 runs of 10 s each, with automatic filter and focus modes. The correlogram quality, intercept, fit error, and count rate were checked from the instrument report for each measurement before data interpretation. All analyses were conducted in triplicate and expressed as mean ± standard deviation (SD).

ζ potential measurements were performed in aqueous medium at 25 °C using the same formulation concentration and without prior dilution. Critical micelle concentration (CMC) and critical micellization temperature (CMT) were not independently determined in the present study; therefore, the DLS results should be interpreted as apparent hydrodynamic sizes of the colloidal populations present under the specific measurement conditions used here, namely 25 °C, 0.5% (w/v) F127, and no dilution.

### 2.9 Atomic Force Microscopy

Atomic force microscopy (AFM) was employed to characterize the surface morphology and local nanomechanical properties of dried F127-based films deposited on mica substrates (Bruker, Santa Barbara, CA, USA), qpBioT probes (Nanosensors) with a nominal spring constant of 0.08 N m^–1^ were employed. The actual spring constant of each probe was calibrated using the thermal noise method prior to measurements. Samples were prepared by depositing the formulation solution onto freshly cleaved mica substrates to form thin films. Measurements were carried out in PeakForce Tapping Quantitative Nanomechanics (QNM) mode, with a resolution of 256 × 256 pixels per scan and a scan frequency of 0.5 Hz. Topographical parameters, including structure size, height, and root mean square roughness (*R*_q_), were evaluated from the height maps.

The roughness parameter (*R*_q_) was calculated according to eq 2:

2 $$R_{q}=\sqrt{\frac{1}{N}\sum_{i=1}^{N}{\left(z_{i}-\mathrm{z̅}\right)}^{2}}$$

where *z*_*i*_ represents the height of each pixel, *z̅* is the mean height of the analyzed area, and *N* is the total number of pixels. The scan area was set to 10 × 10 μm. Prior to roughness analysis, the images were flattened using a third-order polynomial fit.^29^

The apparent lateral dimensions of the surface aggregates were estimated using nanoscope analysis 2.0 software (Bruker) by applying the section analysis tool, in which the width at half-height of each structure was measured (Figure 1B). These values were considered apparent dimensions due to the influence of tip convolution effects on AFM lateral measurements, particularly for soft polymeric nanostructures. The results are reported as mean ± standard deviation (SD).^30^

Adhesion force (*F*_adh_) and Young’s modulus (*E*) were extracted from force–distance curves. The adhesion force was obtained from the retraction curve (Figure 1B) as the minimum cantilever deflection force (*F*_min_), corresponding to the maximum probe–sample interaction.^31^

The apparent DMT modulus was determined by fitting the retraction segment of the force–distance curves using the Derjaguin–Muller–Toporov (DMT) contact model, as described in eq 3:

3 $$F_{tip}=\frac{4}{3}E^{*}\sqrt{R}\delta^{3/2}+F_{adh}$$

where *F*_tip_ is the tip–sample interaction force, E* is the reduced apparent DMT modulus, *R* is the tip radius, δ is the indentation depth, and *F*_adh_ is the adhesion force. Because the measurements were performed on soft polymeric films supported on mica, the reported modulus values should be interpreted as apparent values.^32^

### 2.10 Scanning Electron Microscopy

Sample of FBC was rapidly frozen in liquid nitrogen (−196 °C) and subsequently lyophilized for 24 h using a thermo micro modulyo freeze dryer (Thermo Electron Corporation, Pittsburgh, PA, USA). The lyophilized samples were then sputter-coated using a BAL-TEC SCD 050 sputter coater (Balzers, Liechtenstein). Morphological analysis was performed using a FEI Quanta 250 scanning electron microscope (Thermo Fisher Scientific, Karlsruhe, Germany) at magnifications corresponding to scale bars of 100 and 50 μm.^24^

### 2.11 Cytotoxicity Assay (GM07492A Cell Line)

Cytotoxicity was evaluated using the GM07492A cell line, a non-tumorigenic human lung fibroblast model. Cells were maintained in culture flasks containing Dulbecco’s modified Eagle medium (DMEM) supplemented with 10% fetal bovine serum (FBS) and 1% antibiotic solution (penicillin–streptomycin and amphotericin B), under a humidified atmosphere of 5% CO_2_ at 37 °C.

Upon reaching approximately 60–80% confluence, cells were subcultured to obtain a homogeneous cell suspension for the assays. Briefly, monolayers were washed with phosphate-buffered saline (PBS), followed by enzymatic dissociation using 1× trypsin for 5 min at 37 °C. Trypsin activity was neutralized by the addition of DMEM supplemented with 10% FBS. The cell suspension was centrifuged at 1200 rpm for 10 min and resuspended in 2 mL of supplemented culture medium.

Cell counting was performed using a Neubauer chamber with 0.04% trypan blue solution (1:1, v/v), and the final cell density was adjusted to 2 × 10^6^ cells/mL. Cytotoxicity was assessed using the 3-(4,5-dimethylthiazol-2-yl)-2,5-diphenyltetrazolium bromide (MTT) assay (Invitrogen). Cells were seeded in flat-bottom 96-well plates at a density of 1 × 10^4^ cells per well and incubated for 24 h in DMEM containing 10% FBS under standard conditions (5% CO_2_, 37 °C).^33^ Subsequently, cultures were treated with serial dilutions of the formulations (FB, FC, and FBC) and the vehicle formulation (F), using a final volume of 100 μL per well. The tested concentration range was 500–3.9 μg mL^–1^. These values refer to the nominal concentration of each complete formulation dispersion after dilution in the culture medium, rather than to the isolated concentration of F127, brachydins, or curcumin alone. For the vehicle control (F), the nominal concentration corresponds to the F127- based dispersion without active compounds. For FB, FC, and FBC, the same nominal formulation concentration range was used, while the equivalent concentrations of brachydins and/or curcumin varied according to the formulation composition described in Section 2.4. Therefore, the cytotoxicity assay compares the biological response to the complete formulations under equivalent nominal formulation exposure, not to equal isolated active-compound concentrations. Two experimental conditions were evaluated: with and without light exposure.

All treatments were performed in DMEM supplemented with 1% FBS.

After 24 h of treatment, 10 μL of MTT solution (5 mg/mL) was added to each well, followed by incubation for 3 h at 37 °C to allow for formazan crystal formation. The supernatant was then carefully removed, and 100 μL of 99% ethanol was added to each well to dissolve the crystals.

Absorbance was measured at 570 nm using a microplate spectrophotometer (BioTek Epoch).^34^ Cell viability was expressed as a percentage relative to the negative control (untreated cells), calculated as

4 $$cell\;viability\;\left(\%\right)=\left(\frac{{absorbance}_{treatment}}{{absorbance}_{negative\;control}}\right)\times 100$$

The negative control (NC) consisted of DMEM supplemented with 1% FBS, while dimethyl sulfoxide (DMSO) was used as the positive control (PC). All experiments were performed in triplicate across three independent assays. Data were organized in Microsoft Excel, and statistical analyses were conducted using GraphPad Prism 8 (GraphPad Software Inc., San Diego, CA, USA) to determine mean cell viability values.

### 2.12 Antileishmanial Activity against *L. amazonensis* Promastigotes

The antileishmanial activity of the formulations F, FB, FC, and FBC was evaluated against promastigote forms of *L. amazonensis*. Parasites were prepared at a concentration of 5 × 10^7^ cells mL^–1^, and 10 μL of the suspension was subjected to treatment in the presence or absence of light exposure for 10 min. Following treatment, the parasites were transferred to 96– well plates at a final density of 5 × 10^5^ cells per well in Schneider’s insect medium.

The cultures were then exposed to serial dilutions of the formulations over the concentration range of 500–3.9 μg mL^–1^. As described for the cytotoxicity assay, these values refer to the nominal concentration of each complete formulation dispersion after dilution in the culture medium, rather than to the isolated concentration of F127, brachydins, or curcumin alone. Thus, F was evaluated as the vehicle formulation containing F127 without active compounds, whereas FB, FC, and FBC were evaluated as complete drug-loaded formulation dispersions. The equivalent concentrations of brachydins and/or curcumin in each treatment condition were determined by the composition of each formulation described in Section 2.4. Therefore, comparisons among FB, FC, and FBC were performed under equivalent nominal formulation exposure, not under identical isolated active-compound concentrations.

Pentamidine (PTM) with an IC_50_ of 4 μg/mL was used as the positive control, while Schneider’s medium alone served as the negative control. After 48 h of incubation under appropriate growth conditions, 10 μL of MTT solution (5 mg/mL; 3-(4,5-dimethylthiazol-2-yl)-2,5-diphenyltetrazolium bromide) was added to each well. Plates were then incubated for an additional 3 h at 37 °C to enable the formation of formazan crystals.

Subsequently, 100 μL of 10% dimethyl sulfoxide (DMSO) was added to solubilize the crystals, and absorbance was measured at 570 nm using a microplate spectrophotometer. The half-maximal inhibitory concentration (IC_50_) values were determined by nonlinear regression analysis using GraphPad Prism version 8.0.2 (GraphPad Software Inc., San Diego, CA, USA).

### 2.13 Light Source and Irradiation Conditions

Light-assisted assays were conducted using a blue LED light source assembled from multiple LED arrays mounted on an aluminum metallic base to illuminate 96-well plates from below (Figure 1C,D). The system consisted of six LED plates, each containing 48 blue LEDs, arranged to provide illumination of the wells in the culture plate. The LEDs were connected in parallel and uniformly distributed over the metallic support.

The irradiance of the light source was measured using a spectroradiometer (Ocean Optics USB2000+). The average irradiance measured at the sample surface was 4.65 mW cm^–2^, with the culture plate positioned at a fixed distance of 2.0 cm from the LED source. During irradiation, eutectic ice packs were used in the experimental setup to minimize heating of the irradiation source. Samples assigned to the irradiated groups were exposed to blue light for 10 min, corresponding to a calculated fluence of 2.79 J cm^–2^.

The light dose delivered to the samples was calculated according to eq 5:

5 D=I×t

where *D* is the light dose (J cm^–2^), *I* is the irradiance (W cm^–2^), and *t* is the irradiation time (s).^26^

For the fibroblast cytotoxicity assay, GM07492A cells were seeded in 96- well plates and incubated for 24 h to allow cell attachment, as described in Section 2.11. The culture medium was then replaced with medium containing the corresponding formulation dilution. For irradiated groups, light exposure was performed immediately after formulation addition for 10 min. After irradiation, plates were returned to standard culture conditions and incubated for 24 h, after which the MTT assay was performed. For dark groups, cells received the same formulation dilutions and were maintained under identical conditions but protected from light exposure.

For the antileishmanial assay, *L. amazonensis* promastigotes were exposed to the corresponding formulation dilutions and then irradiated for 10 min in the light-treated groups. After irradiation, parasites were incubated for 48 h under appropriate culture conditions, and parasite viability was then evaluated by the MTT assay, as described in Section 2.12. For dark groups, parasites received the same formulation dilutions and were maintained under identical conditions but protected from light exposure.

For both fibroblast and parasite assays, formulation-only dark controls and formulation-plus-light groups were included. Vehicle controls containing empty F127 micelles (F) were evaluated under both dark and irradiated conditions. Untreated controls maintained in the absence of formulations were used to define baseline viability. Light-only controls consisted of untreated cells or parasites exposed to the same irradiation protocol in the absence of formulations.

The phrase “without prior incubation” refers specifically to the absence of an additional pre-irradiation uptake period between formulation addition and light exposure. Therefore, irradiation was performed after addition of the formulation to the biological system, but without an additional waiting period before light exposure. The available light-source records provide the LED configuration, irradiance, irradiation distance, irradiation time used in the present assays, use of eutectic ice packs to minimize heating of the irradiation source, and calculated fluence. However, LED peak wavelength, spectral bandwidth, irradiance-uniformity mapping across the full 96-well plate, and direct temperature variation at the sample level during irradiation were not fully documented and should be considered methodological limitations of the irradiation protocol.

### 2.14 Statistical Analysis

Data are expressed as mean ± standard deviation (SD), unless otherwise stated. Data distribution was assessed using the Shapiro–Wilk normality test. When data followed a normal distribution, statistical comparisons were performed using analysis of variance (ANOVA), followed by Tukey’s multiple-comparison post hoc test. When normality was not confirmed, the corresponding non-parametric analysis was performed using the Kruskal–Wallis test followed by Dunn’s multiple-comparison test. Statistical significance was considered at *p* < 0.05.

For cytotoxicity and antileishmanial assays, comparisons were performed not only against the positive controls, but also among formulations and between irradiation conditions. For Figure 6, fibroblast viability was compared among formulations at the same nominal concentration and irradiation condition, and dark versus light conditions were compared within each formulation at the same concentration. For Figure 7, parasite viability was compared among formulations at the same nominal concentration and irradiation condition, and dark versus light conditions were compared within each formulation at the same concentration. DMSO and pentamidine were used as assay reference controls, but they were not used as the only basis for evaluating differences among FC, FB, and FBC or between dark and irradiated conditions.

**Figure 2:**
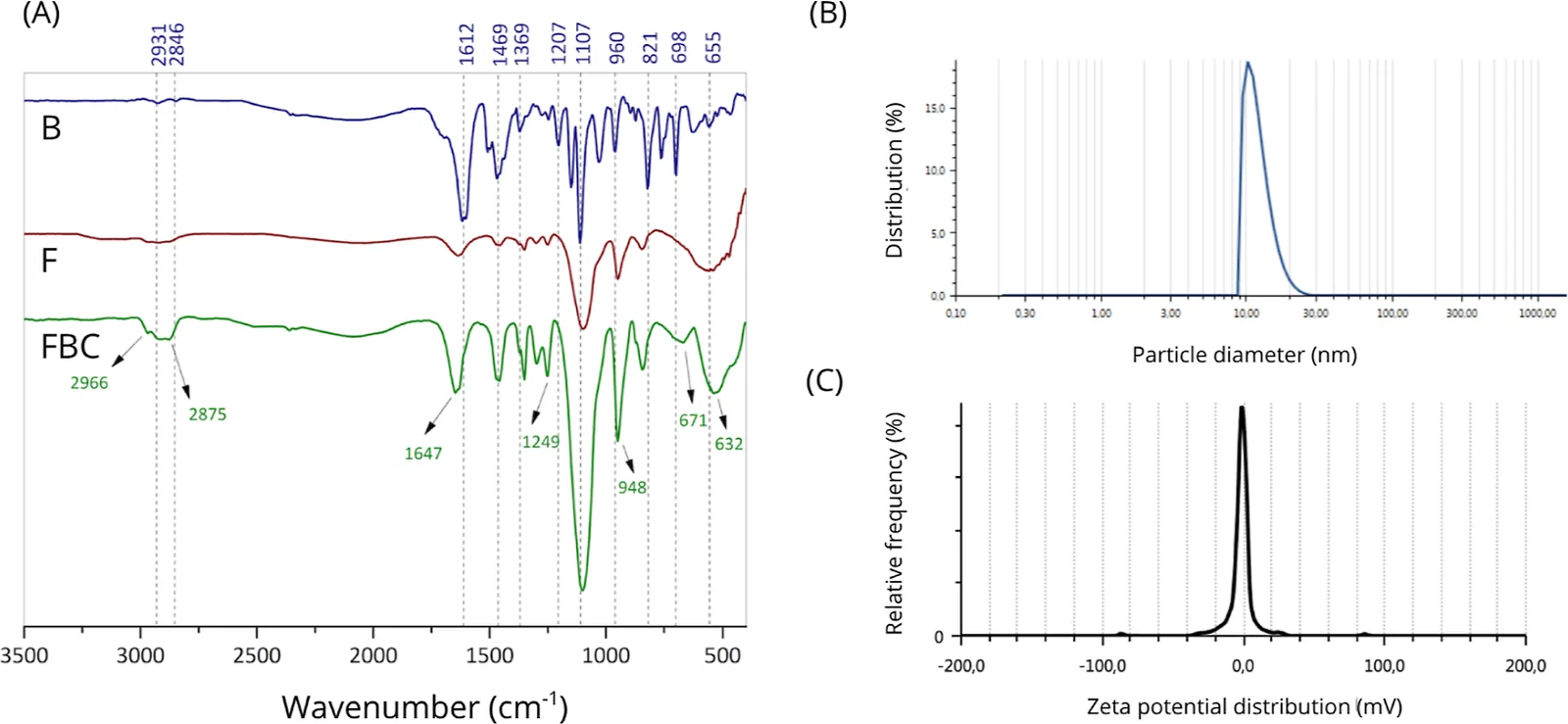
Physicochemical characterization of F127 micelles co-loaded with the brachydin-enriched fraction and curcumin (FBC). (A) FTIR spectra of the brachydin-enriched fraction/curcumin active mixture, empty F127 micelles (F), and the co-loaded F127 formulation (FBC), showing band retention and frequency shifts consistent with changes in the vibrational environment of the active materials after formulation with F127. (B) DLS size distribution of FBC, showing a polydisperse colloidal profile with an apparent mean hydrodynamic diameter (*D*_h_) of 406.5 ± 37.2 nm and a number-weighted smaller population centered at approximately 12.1 nm. (C) Zeta potential distribution of FBC, showing a near-neutral surface charge centered at −0.8 ± 0.1 mV. Measurements were performed at 25 °C.

**Figure 3:**
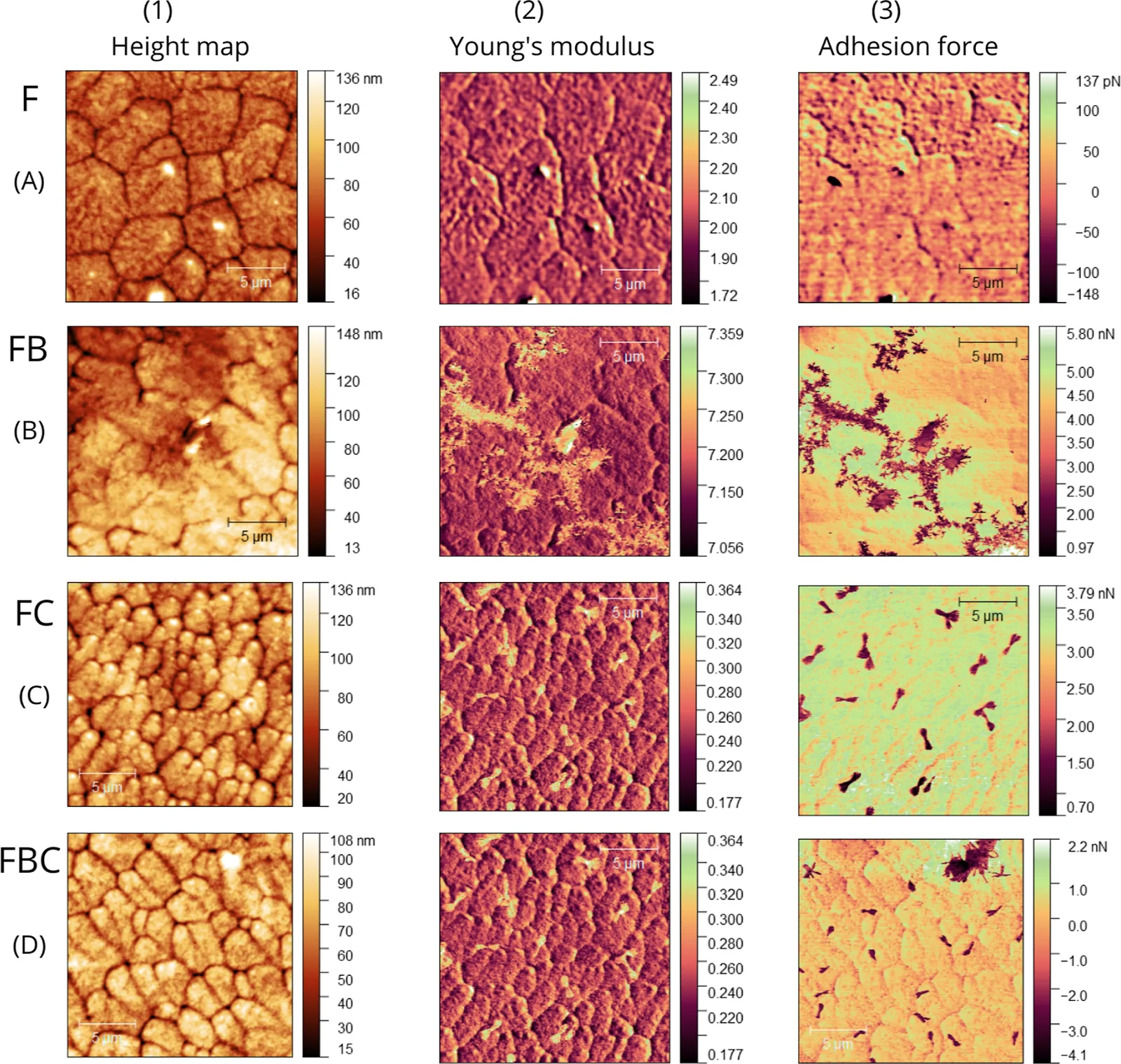
Representative AFM images of the four F127-based formulations deposited on mica and analyzed in PeakForce QNM mode: (A) empty F127 micelles (F), (B) F127 micelles loaded with the brachydin-enriched fraction (FB), (C) F127 micelles loaded with curcumin (FC), and (D) F127 micelles co-loaded with the brachydin-enriched fraction and curcumin (FBC). For each formulation, columns 1,2,3 correspond to the height map, apparent DMT modulus map, and adhesion force map, respectively. The images show the topographical and nanomechanical features of dried/deposited films and should not be interpreted as direct images of hydrated micelles in aqueous dispersion. Formulation-dependent differences in surface organization and local tip–sample interactions can be observed among the systems. Color scales represent height (nm), apparent DMT modulus (MPa), and adhesion force (nN or pN, as indicated in each map). Scale bar: 5 μm.

**Figure 4:**
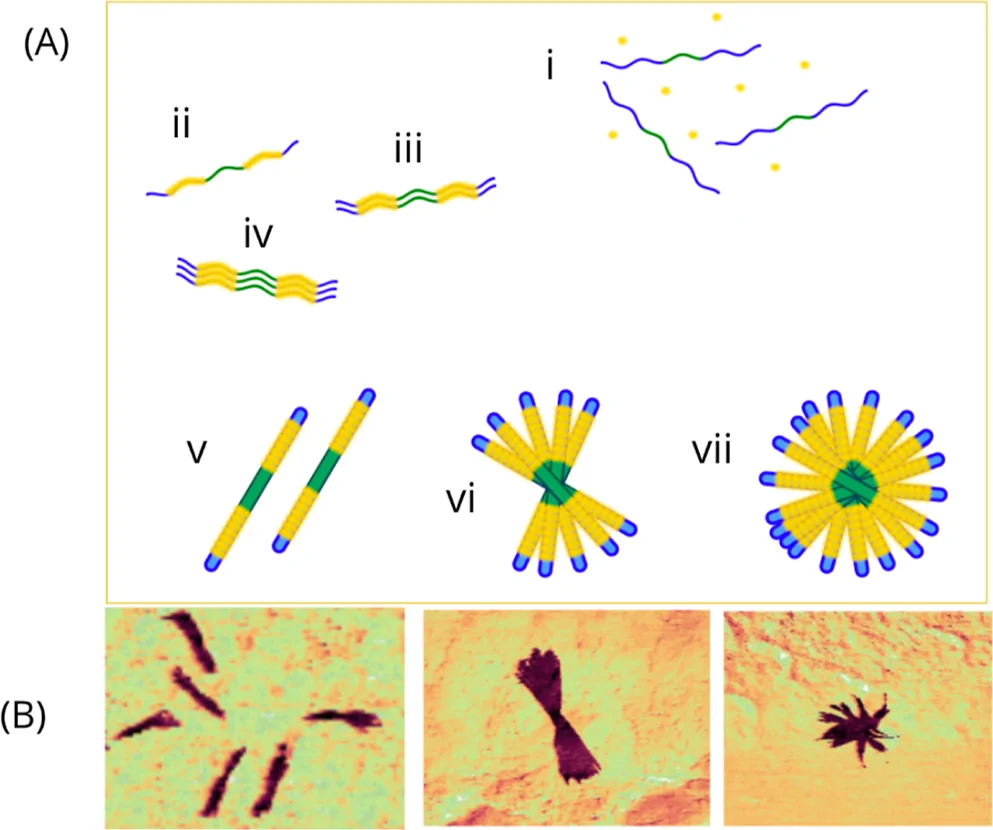
Hypothetical self-assembly model for FBC micelles. (A) Schematic representation of a plausible organization process involving dispersion of F127 copolymer chains in aqueous medium, possible association of the hydrophobic active materials, namely the brachydin-enriched fraction and curcumin, with PPO-rich domains through non-covalent interactions, and subsequent formation of micellar or higher-order assemblies. (B) Representative AFM images showing elongated, clustered, and radially organized surface features observed after sample deposition and drying. This model is intended as an interpretative scheme consistent with FTIR, DLS, and AFM observations and should not be interpreted as direct structural determination of the internal micellar architecture. Interactions such as hydrophobic association and possible aromatic stacking are proposed as potential contributors to active-material incorporation, but should not be assigned to purified individual brachydins without further compound-specific structural evidence.

**Figure 5:**
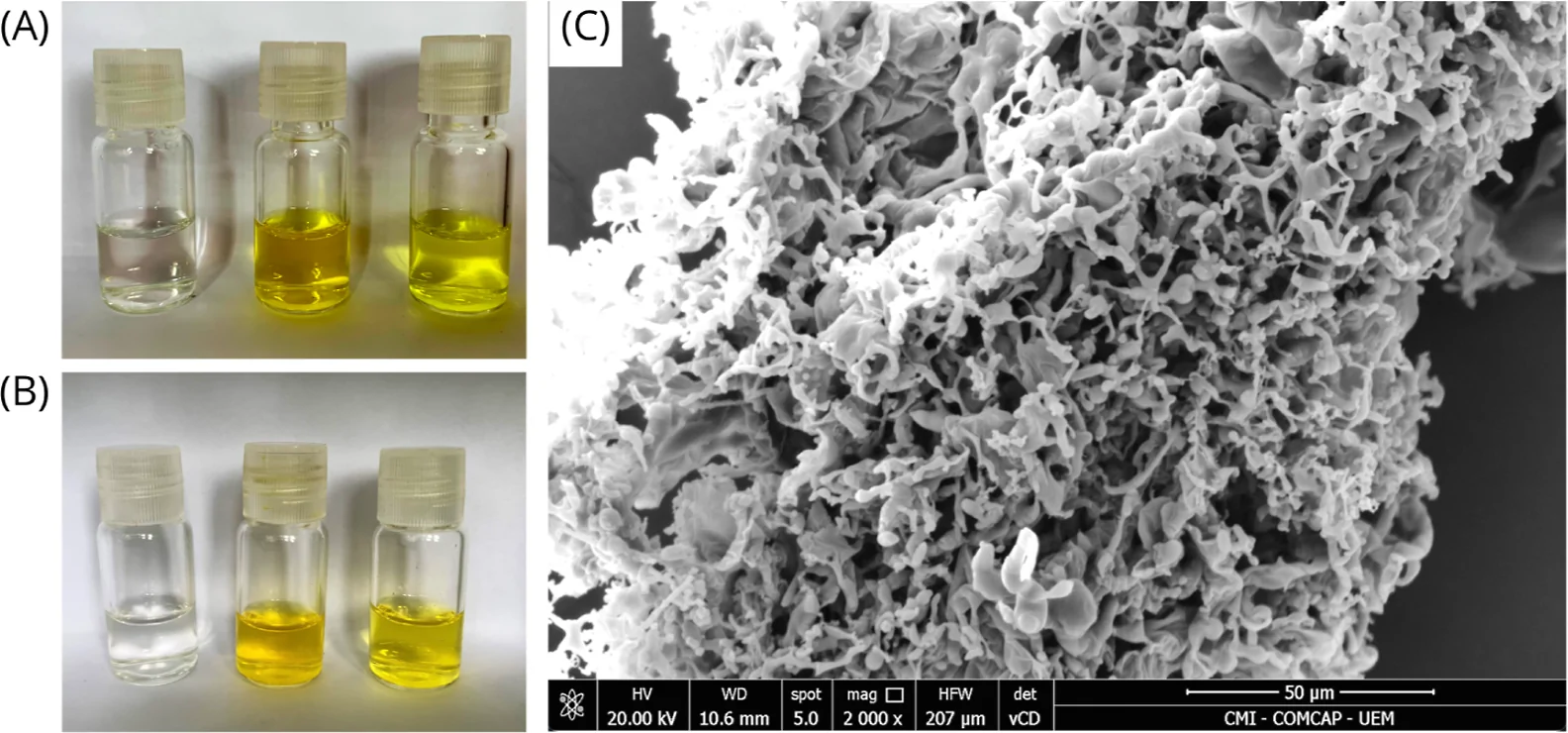
Macroscopic and SEM-based evaluation of F127 formulations. (A) Representative images of FB, FC, and FBC formulations after 72 h of preparation. (B) Representative images of the same formulations after 30 days of storage. (C) SEM micrograph of lyophilized FBC after 30 days of storage, showing a micrometer-scale porous and interconnected dried morphology with fibrillar-like domains. The SEM image represents the processed dried material after freezing, lyophilization, drying, and sputter-coating, and should not be interpreted as direct evidence of hydrated micelles or hierarchical micellar networks in aqueous dispersion. The photographs in panels A and B were taken by the authors during the present study, and the SEM micrograph in panel C was generated as part of the present study; none of these images has been previously published.

**Figure 6:**
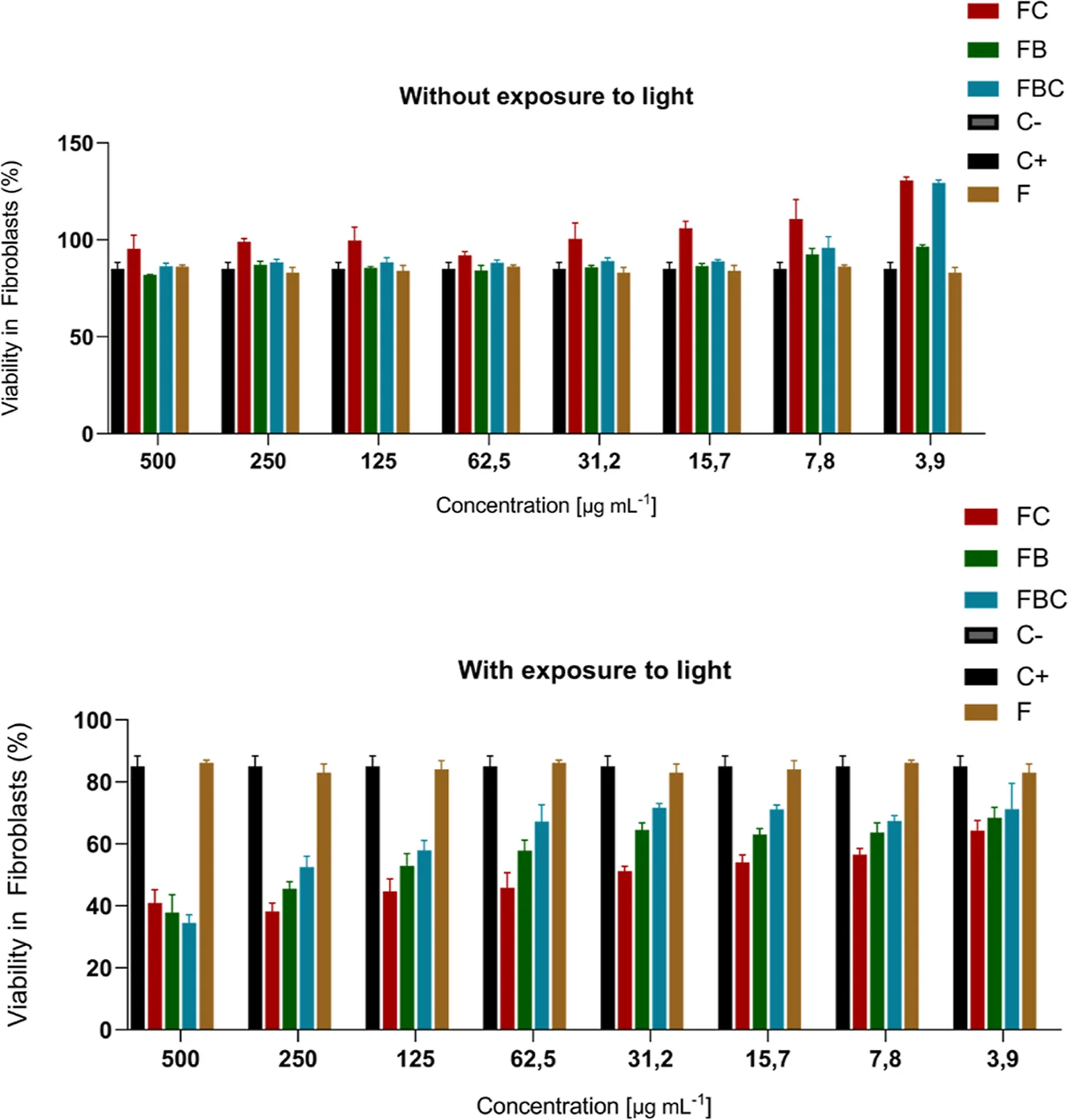
In vitro cytotoxicity of FC, FB, and FBC formulations in GM07492A human lung fibroblasts under dark and light exposure conditions. Cells were treated with increasing nominal formulation concentrations (3.9–500 μg mL^–1^) for 24 h, and viability was assessed by the MTT assay. Data are expressed as percentage of viable cells relative to the untreated control. Results are presented as mean ± standard deviation (SD) from three independent experiments performed in triplicate, with a 95% confidence interval. Statistical analysis was performed as described in Section 2.14. Comparisons included differences among formulations at the same nominal concentration and irradiation condition, as well as dark versus light comparisons within each formulation at the same concentration. DMSO was used as the positive cytotoxicity control. Statistical significance was considered at *p* < 0.05. The concentration-dependent viability profile should be considered when interpreting mammalian-cell safety, particularly under light exposure at higher formulation concentrations.

**Figure 7:**
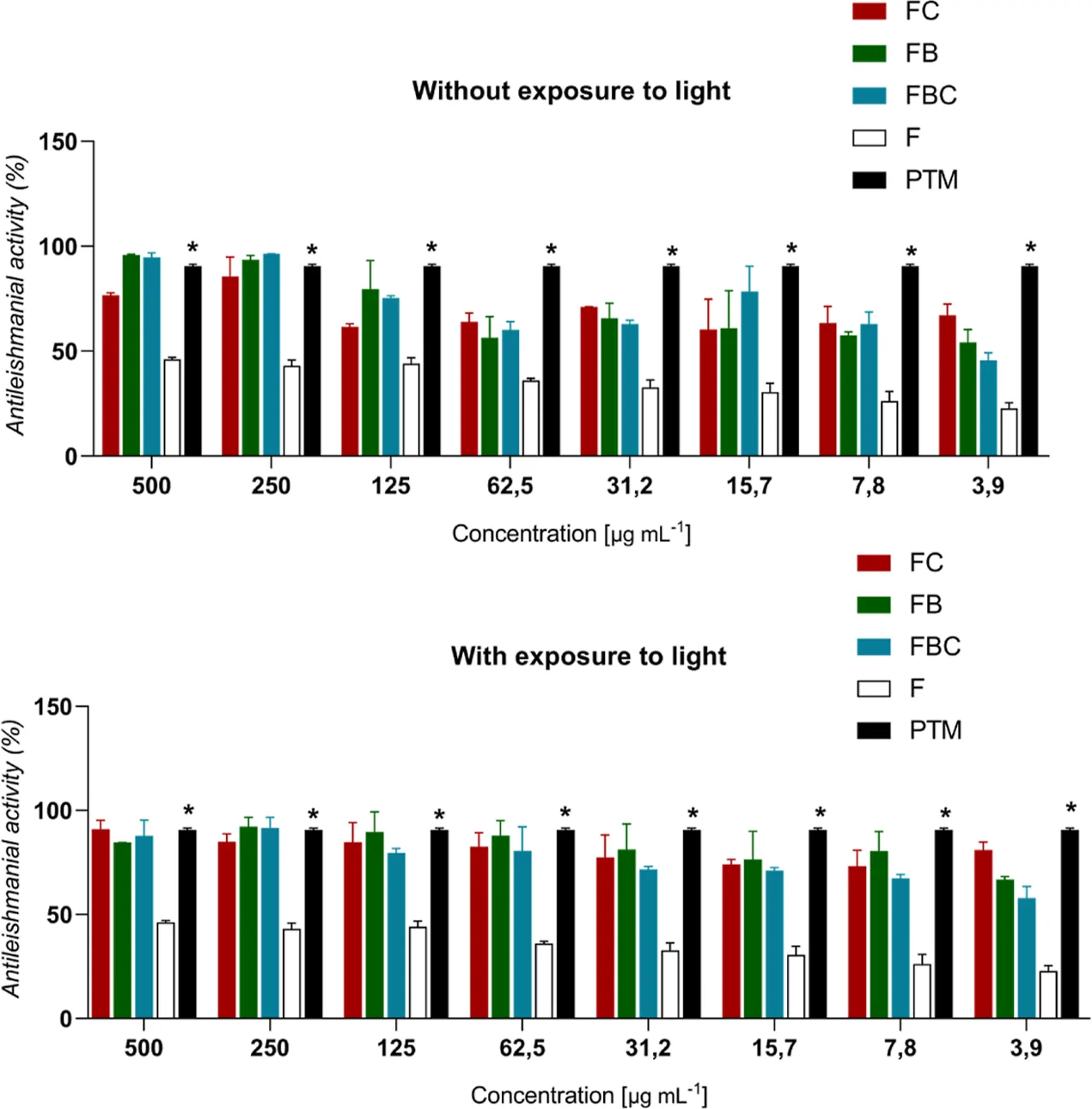
In vitro antileishmanial activity of FC, FB, and FBC formulations against *L. amazonensis* promastigotes under dark and light exposure conditions. Parasites were treated with increasing nominal formulation concentrations (3.9–500 μg mL^–1^) for 48 h, and the biological response was determined by the MTT assay. Data are expressed as percentage of antileishmanial activity relative to the untreated negative control. Results are presented as mean ± standard deviation (SD) from three independent experiments performed in triplicate, with a 95% confidence interval. Statistical analysis was performed as described in Section 2.14. Comparisons included differences among formulations at the same nominal concentration and irradiation condition, as well as dark versus light comparisons within each formulation at the same concentration. Pentamidine (PTM) was used as the reference antileishmanial control. Statistical significance was considered at *p* < 0.05; when indicated, *p < 0.001 denotes highly significant differences.

IC_50_ and CC_50_ values were determined by nonlinear regression analysis using concentration–response curves. Statistical analyses and graphical representations were performed using Origin 2021 software (OriginLab Corporation, Northampton, MA, USA) and/or GraphPad Prism version 8.0.2 (GraphPad Software Inc., San Diego, CA, USA), according to the analysis performed. For nanomechanical measurements, one adhesion force and one apparent DMT modulus value were extracted from each force–distance curve, generating maps with a resolution of 256 × 256 pixels. These pixel-level data were used to construct representative maps and value distributions. However, because force curves acquired within the same map are spatially correlated, they were not considered independent experimental replicates. For statistical analysis, the mean value obtained from each independent AFM map was used as the experimental unit. Five independent maps were analyzed per sample (*n* = 5). For roughness analysis, one *R*_q_ value was calculated per height map, and statistical comparisons were performed using the same map-level approach. All results were expressed as mean ± standard deviation (SD).

## 3 Results and Discussion

### 3.1 Preliminary Stability Assay

The structural integrity of BC-loaded F127 micelles was initially evaluated through visual analysis. First, the ability of F127 copolymeric nanoparticles to solubilize the natural compounds during the hydration step was investigated at room temperature using concentrations of 60 μg mL^–1^ of brachydins and 74 μg mL^–1^ of curcumin.

These concentrations were selected based on previously reported IC_50_ values.^24^ Under these conditions, BC could not be effectively solubilized, even when the temperature was increased to 60 °C, mainly due to the poor solubility of curcumin. A subsequent experiment was conducted using a reduced curcumin concentration, adjusting BC levels to 60 and 37 μg mL^–1^ for brachydins and curcumin, respectively, while maintaining room temperature during the hydration step.

No precipitation was observed immediately after preparation; however, after 72 h, the formulation prepared at room temperature exhibited visible loss of macroscopic homogeneity, characterized by the formation of filamentous precipitates. In a further experiment, the effect of hydration temperature on the preliminary macroscopic behavior of the formulation was evaluated. The concentrations of the brachydin-enriched fraction and curcumin were maintained at 60 and 37 μg mL^–1^, respectively, and the hydration temperature was increased to 60 °C.

Under these conditions, the formulation showed no visible precipitation or phase separation after 72 h. Based on this preliminary visual screening, hydration at 60 °C was selected for preparation of the co-loaded F127 formulation, designated as FBC (Figure 1). These observations should be interpreted as evidence of improved short-term macroscopic homogeneity during the preliminary formulation screening, rather than as quantitative proof of physical or colloidal stability.

### 3.2 Accelerated Stability Testing

According to the United States Pharmacopeia (USP), the stability of active pharmaceutical ingredients (APIs) requires the evaluation of both chemical and physical stability over time, and any physical changes in the dosage form should be reversible prior to administration (e.g., by shaking).^27,28^

In terms of chemical stability, a formulation is considered stable if the API concentration remains within 90–110% of its initial value (day 0). Accelerated stability testing was performed by subjecting FBC to alternating temperature cycles of 24 h at room temperature (25 ± 3 °C) and 24 h under controlled refrigeration (5 ± 3 °C).

After seven thermal cycles, the active-material content detected by HPLC-PDA remained above 90% of the initial value, indicating preservation of most of the detectable hydrophobic active material under the tested conditions. However, because the present study did not include a complete compound-specific stability dataset reporting separate mean ± SD values for curcumin and individual brachydin-related peaks after storage, this result should be interpreted as evidence of apparent active-material retention rather than as a fully resolved quantitative stability profile for each chemical constituent.

Regarding physical stability, no changes in color or odor were detected, and no visible precipitation or phase separation occurred throughout the evaluated period (see Figure 5). These observations indicate macroscopic physical integrity under the tested conditions. Nevertheless, visual inspection does not provide direct information on nanoscale colloidal stability, such as time-dependent changes in hydrodynamic diameter (*D*_h_), PDI, scattering intensity, or ζ potential.

Therefore, the stability results should be interpreted cautiously as evidence of short-term apparent active-material retention and macroscopic physical integrity, rather than as definitive proof of chemical and colloidal stability. Although the detectable active-material content remained above 90% after the seventh thermal cycle, the evolution of micellar size distribution and surface charge during storage was not monitored. This is an important limitation because Pluronic-based micellar systems may undergo concentration- and temperature-dependent reorganization, aggregation, or changes in size distribution over time.

Overall, the accelerated stability assay supports the short-term preservation of the detectable active material and the absence of visible macroscopic instability under the tested conditions. However, the statement that FBC is chemically and physically stable for at least 14 days was moderated. A more rigorous stability assessment would require quantitative time-dependent HPLC-PDA analysis of curcumin and brachydin-related peaks, reported as mean ± SD, together with DLS monitoring of *D*_h_, PDI, scattering intensity, and ζ potential before and after storage.

### 3.3 Encapsulation Efficiency (EE %)

The encapsulation efficiency (EE %) was evaluated as an apparent formulation-level parameter reflecting the amount of hydrophobic active material retained in the centrifuged and filtered liquid formulation after preparation. As described in Section 2.5, samples were centrifuged at 3000 rpm for 30 min and filtered through a 0.2 μm membrane before HPLC-PDA analysis. Therefore, the EE % value represents the recovery of active material remaining in the liquid formulation after removal of non-dispersed or precipitated material, rather than a strict separation between molecularly free soluble compounds and micelle-associated compounds.

For FBC, the nominal amount of active material initially added corresponded to the sum of the brachydin-enriched fraction and curcumin. In a standard 10 mL batch, this represented 0.60 mg of brachydin-enriched fraction plus 0.37 mg of curcumin, totaling 0.97 mg of hydrophobic active material, together with 50.0 mg of F127. The apparent EE % calculated for FBC was 92.65 ± 0.48%, corresponding to an estimated recovered active-material mass of approximately 0.90 mg in the centrifuged and filtered formulation. Based on this value, the apparent active-material loading corresponded to approximately 1.8% relative to the F127 mass.

Because the brachydin component corresponds to an enriched dichloromethane fraction rather than to purified individual compounds, separate EE % values for brachydin A, brachydin B, and brachydin C were not calculated in the present manuscript. In addition, because the method used here was based on centrifugation, filtration, and HPLC-PDA analysis of the recovered liquid formulation, a complete compound-specific mass balance distinguishing free soluble, micelle-associated, precipitated, and filter-retained fractions was not established. Thus, the EE % value should be interpreted as an apparent recovery/incorporation parameter for the co-loaded formulation, not as a fully resolved compound-specific encapsulation efficiency.

This result nevertheless indicates that most of the hydrophobic active material initially added to the FBC formulation remained in the liquid phase after preparation, centrifugation, and filtration. Therefore, the use of 0.5% (w/v) F127 was sufficient to maintain a high apparent recovery of the brachydin-enriched fraction and curcumin under the selected preparation conditions. Future studies should include compound-specific calibration, recovery, LOD/LOQ determination, and mass-balance analysis to quantify separately the encapsulation and loading behavior of curcumin and individual brachydin-related peaks.

### 3.4 FTIR Analysis

FTIR analysis was used as a complementary technique to evaluate possible changes in the vibrational environment of the hydrophobic active materials after formulation with Pluronic F127. In this section, BC is used operationally to refer to the active mixture composed of the brachydin-enriched fraction and curcumin, rather than to a chemically pure compound or a single molecular entity. Therefore, the FTIR results should be interpreted as indirect spectral evidence of formulation-induced changes in the local environment of this active mixture, and not as a compound-specific purity analysis, a definitive confirmation of encapsulation, or direct proof of molecular dispersion within the micellar core.

The absorption band centered at 1612 cm^–1^ in the BC spectrum, assigned mainly to conjugated C=C stretching vibrations present in aromatic and polyphenolic structures, shifted to 1647 cm^–1^ in the FBC formulation. In addition, bands associated with aromatic ring vibrations, including C=C stretching and C–H out-of-plane bending modes at 1469, 821, and 689 cm^–1^, were still observed after formulation. These spectral features suggest that the main vibrational signatures of the active mixture were retained under the preparation conditions, with no evident disappearance of the major bands analyzed. However, FTIR alone cannot demonstrate complete preservation of chemical integrity or exclude subtle degradation, isomerization, or changes in the relative composition of the brachydin-enriched fraction.^35,36^

The observed shifts in vibrational frequencies are consistent with changes in the local electronic and hydrogen-bonding environment of functional groups present in the active mixture after contact with the F127 matrix. Such changes may arise from non-covalent interactions between hydrophobic and aromatic domains of the active materials and PPO-rich regions of F127, as previously discussed for Pluronic-based systems containing hydrophobic natural products.^21,22^ Nevertheless, these assignments should be considered tentative. FTIR does not directly resolve the location of the active materials within the micelles and cannot, by itself, confirm incorporation specifically into the PPO core, molecular-level dispersion, or a defined internal micellar architecture.

The shift of the broad band observed in the F127 spectrum from 655 to 632 cm^–1^ in the FBC formulation is also consistent with changes in the vibrational environment of oxygen-containing groups and possible modification of intermolecular hydrogen-bonding patterns. However, this observation should not be interpreted as conclusive evidence of a specific interaction mechanism. Because the present FTIR comparison did not include a complete set of additional controls, such as physical mixtures, pure curcumin, the isolated brachydin-enriched fraction, FB, and FC under identical acquisition and processing conditions, the spectral interpretation remains qualitative and supportive.

Overall, the FTIR data are consistent with the association of the hydrophobic active materials with the F127-based system through non-covalent interactions, but they do not conclusively demonstrate encapsulation, molecular dispersion, preservation of complete chemical integrity, or localization within PPO-rich micellar domains. Additional techniques, such as DSC, XRD, fluorescence spectroscopy, NMR, release/partitioning assays, or a systematic FTIR comparison including physical mixtures and single-loaded formulations, would be necessary to strengthen molecular-level claims. Accordingly, the FTIR results are interpreted here as complementary evidence supporting formulation-dependent changes in the vibrational environment of the brachydin-enriched fraction/curcumin active mixture after incorporation into the F127-based formulation.

### 3.5 Particle Size and ζ Potential

The particle size and ζ potential of the F127-based formulations were evaluated by dynamic light scattering (DLS) in aqueous medium. The FBC formulation showed an apparent mean hydrodynamic diameter (*D*_h_) of 406.5 ± 37.2 nm under the measurement conditions used in this study (Figure 2B), with a PDI of approximately 0.292. In addition, the number-weighted size distribution showed a smaller population centered at approximately 12.1 nm. These results indicate that FBC should be interpreted as a polydisperse F127-based colloidal formulation, in which smaller nanosized assemblies coexist with larger colloidal populations or aggregates. Therefore, the DLS data should not be interpreted as evidence of a single uniform micellar population or as definitive proof of a specific internal micellar architecture.

The PDI value of approximately 0.292 indicates moderate polydispersity rather than a narrow or highly homogeneous size distribution. Thus, FBC should be described as a polydisperse F127-based formulation containing both smaller nanosized assemblies and larger colloidal populations. This behavior is consistent with Pluronic-based micellar systems containing hydrophobic bioactive materials, in which micelle reorganization, transient association, and formulation-dependent aggregate contributions may coexist in aqueous medium.^21,22^

The near-neutral ζ potential value of FBC (−0.8 ± 0.1 mV) is consistent with the nonionic character of Pluronic F127 and the predominance of hydrated PEO chains at the interface. However, because electrostatic stabilization is limited in nonionic F127 systems, colloidal behavior should be interpreted mainly in terms of steric stabilization and polymer hydration rather than surface charge repulsion. In addition, the DLS results were obtained at a single post-preparation time point; therefore, they do not demonstrate time-dependent colloidal stability during storage.

A comparative analysis with previously reported systems (Table 1) highlights formulation-dependent differences in apparent hydrodynamic size and aggregation behavior.

**Table 1:** Comparison of DLS Parameters for F127-Based Systems^a^

system	*D*_h_ (nm)	PDI	ζ (mV)	references
F	18.1 ± 1.0	0.189 ± 0.004	–9.2 ± 1.2	^24^
FB	157.5 ± 13.7	0.281 ± 0.023	–13.0 ± 0.7	^24^
FC	16 ± 2	0.57 ± 0.11	–2.93 ± 0.92	^37^
FBC	406.5 ± 37.2	0.292 ± 0.029	–0.8 ± 0.1	present study

a For FBC, the value of 12.1 nm corresponds to a number-weighted size-distribution peak, whereas the reported *D*_h_ corresponds to the apparent mean hydrodynamic diameter obtained from the included DLS measurements.

In Table 1, F and FBC correspond to formulations measured in the present study using the dispersions as prepared, without dilution, at 25 °C and 0.5% (w/v) F127. Values are reported as mean ± SD when available. FB is included as a previously reported F127 formulation containing a brachydin-enriched fraction and analyzed under comparable formulation conditions.^24^ In contrast, FC micelles are included only as a contextual literature comparator because they correspond to curcumin-loaded Pluronic F127 micelles reported by Klahan et al.^37^ and were not prepared or measured under the same experimental conditions used in the present study. Therefore, comparisons involving FC should be interpreted qualitatively. Importantly, PDI values around or above 0.3 indicate moderate to broad polydispersity rather than a narrow or highly homogeneous size distribution.

The interpretation of the DLS results should consider the temperature—and concentration-dependent micellization behavior of Pluronic F127 and related PEO–PPO–PEO nanocarriers. These systems are known to form dynamic assemblies whose apparent hydrodynamic size may vary according to polymer concentration, temperature, hydration conditions, drug loading, and the balance between individual micelles and transient aggregates.^18–20,23^ In the present study, the formulations were hydrated at 60 °C during preparation to favor solubilization and incorporation of the hydrophobic compounds, whereas DLS measurements were performed at 25 °C using the formulations as prepared, without dilution, at a final F127 concentration of 0.5% (w/v). Thus, the colloidal state detected by DLS at 25 °C represents the equilibrated dispersion under the measurement conditions and may differ from the transient organization present during the heated hydration step.

Although the critical micelle concentration (CMC) and critical micellization temperature (CMT) were not experimentally determined in the present study, the detection of nanosized populations for F, FC, and FBC indicates that F127-based assemblies were present under the measurement conditions. Nevertheless, the absence of direct CMC/CMT determination is a limitation and prevents a definitive description of the micellization boundary or aggregation state of the system. For this reason, the DLS results are interpreted as apparent hydrodynamic sizes of the colloidal populations present at 25 °C and 0.5% (w/v) F127, rather than as a complete description of the micellization process.

The relationship between the empty and loaded systems should also be interpreted within the broader context of F127 micellar carriers for poorly water-soluble compounds. Empty F127 micelles previously exhibited a hydrodynamic diameter of 18.1 ± 1.0 nm, whereas the present FBC formulation showed an apparent mean hydrodynamic diameter of 406.5 ± 37.2 nm. In addition, the number-weighted size-distribution peak at approximately 12.1 nm suggests the coexistence of smaller nanosized assemblies within a broader polydisperse colloidal system. Therefore, the DLS results should not be interpreted as evidence of a uniformly small micellar population, but rather as apparent hydrodynamic behavior influenced by scattering intensity, hydration, intermicellar association, and the relative contribution of smaller assemblies and larger colloidal populations or aggregates.

Previous studies with Pluronic-based systems containing hydrophobic natural products, particularly curcumin, have shown that incorporation into F127 assemblies can improve aqueous dispersion while also modifying aggregation behavior and apparent particle-size distributions.^21,22^ Therefore, the size range observed for FBC should not be viewed as unexpected in isolation, but rather as part of the formulation-dependent behavior commonly observed for PEO–PPO-based carriers loaded with poorly water-soluble bioactive materials. In this sense, the present findings are consistent with the established role of F127 as a solubilizing and dispersing platform, while extending its application to the co-loading of a brachydin-enriched fraction and curcumin for light-assisted antileishmanial evaluation.

In contrast, F127 systems loaded with the brachydin-enriched fraction previously exhibited hydrodynamic diameters of *D*_h_ = 157.5 ± 13.7 nm.^24^ In the present study, FBC showed a larger apparent mean hydrodynamic diameter (*D*_h_ = 406.5 ± 37.2 nm), indicating a greater contribution of larger colloidal populations or aggregates in the co-loaded formulation. At the same time, the number-weighted size-distribution peak at approximately 12.1 nm suggests the presence of smaller nanosized assemblies within the broader polydisperse colloidal system.

This comparison indicates that the simultaneous incorporation of the brachydin-enriched fraction and curcumin altered the association behavior of the F127 assemblies under the measurement conditions, but does not support the interpretation of FBC as a uniformly small micellar population. Because DLS does not resolve the internal architecture of the particles, because F127 aggregation is sensitive to temperature and concentration, and because the brachydin component corresponds to an enriched fraction rather than purified individual compounds, this result should be interpreted as evidence of formulation-dependent colloidal organization rather than definitive proof of an optimized compound-specific co-encapsulation mechanism.

The slight asymmetry in the size distribution, with a tail toward larger diameters, indicates the presence of minor populations of loosely associated aggregates. This behavior is consistent with the dynamic equilibrium between individual micelles, drug-loaded assemblies, and transient aggregates commonly observed in block copolymer systems.^18,20^ These features are also compatible with AFM observations, where nanoscale domains coexist with occasional larger surface assemblies; however, AFM and DLS provide complementary but indirect information and should not be interpreted as definitive evidence of a single uniform micellar population.

The surface charge of FBC was further evaluated by ζ potential measurements (Figure 2C), which revealed a near-neutral value centered at −0.8 ± 0.1 mV. This low absolute ζ potential indicates that electrostatic repulsion is unlikely to be the main mechanism preventing particle association. Therefore, the ζ potential value alone should not be interpreted as direct evidence of colloidal stability.

For Pluronic F127-based systems, stabilization may occur predominantly through steric effects associated with the hydrated poly­(ethylene oxide) (PEO) corona, which can reduce extensive particle–particle association even when electrostatic repulsion is weak.^18,20,23^ In the present study, this interpretation is supported indirectly by the absence of visible precipitation or phase separation during the evaluated stability assays, rather than by the ζ potential value itself.^24^ Nevertheless, the present data do not provide direct evidence of long-term colloidal stability at the nanoscale. Time-dependent DLS measurements, including changes in *D*_h_, PDI, scattering intensity, and ζ potential during extended storage, would be required to confirm colloidal stability more rigorously. Thus, the near-neutral ζ potential should be interpreted as compatible with a sterically stabilized Pluronic-based dispersion, but not as definitive proof of long-term colloidal stability.

Overall, under the measurement conditions used in this study, namely 25 °C, 0.5% (w/v) F127, and no dilution, the DLS results indicate that FBC presents a polydisperse colloidal profile, with an apparent mean hydrodynamic diameter of 406.5 ± 37.2 nm and a number-weighted smaller population centered at approximately 12.1 nm. These data should be interpreted as apparent hydrodynamic behavior under defined experimental conditions rather than as definitive evidence of a unique micellar architecture. Because CMC and CMT were not independently determined and because F127 micellization is strongly dependent on temperature and concentration, comparisons among F, FB, FC, and FBC should be understood as formulation-dependent differences in colloidal populations measured under specific conditions, not as a complete description of the micellization pathway. The DLS profile therefore supports the presence of coexisting smaller nanosized assemblies and larger colloidal populations or aggregates, but it does not imply a highly homogeneous population or definitive proof of an optimized co-encapsulation mechanism.

The relationship between DLS, AFM, and SEM should therefore be interpreted considering the different physical states probed by each technique. DLS measures the hydrodynamic behavior of hydrated colloidal species in aqueous dispersion, whereas AFM and SEM evaluate deposited or dried materials. Consequently, the apparent mean *D*_h_ of approximately 406.5 nm and the number-weighted smaller population around 12.1 nm detected by DLS should not be directly equated with the larger surface domains or interconnected structures observed by AFM and SEM. Instead, these techniques provide complementary information at different levels of organization: DLS supports the presence of a polydisperse hydrated colloidal system in aqueous dispersion, AFM reveals formulation-dependent surface organization and nanomechanical heterogeneity after deposition, and SEM shows the morphology of the dried matrix after freezing and lyophilization. The larger features observed by AFM and SEM may arise from the association, clustering, spreading, collapse, or rearrangement of hydrated colloidal assemblies during substrate deposition, freezing, drying, or lyophilization.^38–40^

Thus, the combined interpretation of DLS, AFM, and SEM supports the formation of an organized F127-based colloidal/dried-state system, but does not establish a direct one-to-one correspondence between the hydrodynamic diameter measured in solution and the dimensions of dried structures observed microscopically. This distinction was included to avoid overinterpreting the microscopic images as direct representations of the native colloidal state.

### 3.6 Atomic Force Microscopy Analysis

AFM analysis was performed to investigate the topographical and nanomechanical properties of F127-based formulations, including height distribution, adhesion force (*F*_adh_), and apparent DMT modulus (E*). Representative AFM images are presented in Figure 3.

AFM analysis provided insight into the topographical and nanomechanical properties of the F127-based formulations after deposition on mica. Therefore, these data should be interpreted as complementary surface-level information rather than as a direct representation of the hydrated colloidal species measured by DLS. Height maps revealed clear formulation-dependent differences (Figure 3). The F formulation [Figure 3A­(1)] exhibited higher surface irregularity, whereas FB [Figure 3B­(1)] and FC [Figure 3C­(1)] showed intermediate behavior. Notably, FBC [Figure 3D­(1)] displayed lower height variations, suggesting a more compact organization of the deposited film. This trend indicates that brachydins and curcumin may contribute to surface reorganization after sample deposition, with a more pronounced effect when both compounds are combined.^22,24^

The high vertical resolution of AFM enables nanoscale structural characterization,^38^ while PeakForce QNM allows simultaneous acquisition of topographical and nanomechanical data, which is particularly relevant for soft polymeric systems. The apparent DMT modulus was determined using the Derjaguin–Muller–Toporov model (eq 3); however, because the measurements were performed on soft films supported on mica, the values should be interpreted as apparent and mainly used for comparative purposes.^41^

Nanomechanical maps [Figure 3A­(2)–D­(2)] showed formulation-dependent mechanical contrast within the deposited films. These contrasts indicate heterogeneity in the dried F127-based matrix after sample deposition on mica, but they should not be interpreted as direct evidence of micellar architecture in aqueous dispersion. Because the measurements were performed on soft dried films supported on a solid substrate, the apparent DMT modulus values should be interpreted as comparative surface-level parameters rather than absolute mechanical properties of hydrated micelles.^22,24^

The apparent modulus and adhesion-force maps suggest that the incorporation of the brachydin-enriched fraction and/or curcumin modifies the local nanomechanical response of the deposited material. However, the qualitative color maps alone do not allow a definitive ranking of stiffness among the formulations, particularly without quantitative values reported as mean ± SD and appropriate statistical analysis. Therefore, the statement that FBC displays the highest apparent modulus was removed. Instead, the AFM data are interpreted as indicating formulation-dependent differences in the organization and surface interactions of dried/deposited films.

Adhesion force maps [Figure 3A­(3)–D­(3)] also revealed formulation-dependent differences in tip–sample interactions. These differences may reflect changes in surface composition, local packing, or drying-induced organization of the F127-based films, consistent with the interpretation of AFM data as surface-level information obtained from deposited samples.^24^ Nevertheless, they should not be used as direct evidence of compact micellar cores, PPO-domain organization, or colloidal stabilization mechanisms in solution. Accordingly, AFM findings were interpreted together with DLS as complementary but distinct information: DLS describes the apparent hydrodynamic behavior of the aqueous dispersion, whereas AFM describes the morphology and nanomechanical response of deposited films after solvent removal.

It is important to emphasize that the AFM data presented here are representative surface maps and were not used to establish a complete quantitative nanomechanical ranking among the formulations. Quantitative roughness, aggregate-size distribution, adhesion force, and apparent DMT modulus values with mean ± SD and statistical comparison were not obtained for all formulations in a way that would support definitive statistical conclusions. Therefore, the AFM results should be interpreted qualitatively as evidence of formulation-dependent differences in the morphology and local nanomechanical response of dried/deposited films, rather than as quantitative proof of superior stiffness, compactness, or structural organization of FBC.

Based on the combined FTIR, DLS, and AFM results, a hypothetical self-assembly model is proposed for the FBC formulation (Figure 4). The hydrophobic active materials present in FBC, namely the brachydin-enriched fraction and curcumin, may interact with F127 through non-covalent associations, including hydrophobic interactions and possible aromatic contributions.^22^ However, the preferential localization of these materials within PPO-rich domains cannot be directly confirmed from the present data. Because the brachydin component corresponds to an enriched fraction rather than to purified individual compounds, these interactions should be interpreted as formulation-level associations involving the active mixture, rather than as compound-specific interactions assigned to isolated brachydin A, B, or C. FTIR band shifts are consistent with changes in the local vibrational environment of the active materials after formulation with F127, but they should be regarded as indirect and supportive evidence rather than definitive proof of encapsulation, molecular dispersion, or a specific internal micellar arrangement.^21,42^

Upon hydration, these interactions may favor the formation of primary micellar assemblies, in which the hydrophobic active mixture is likely accommodated within PPO-rich domains, while PEO chains extend into the aqueous phase and contribute to steric stabilization. The presence of the brachydin-enriched fraction and curcumin may also influence copolymer packing and promote deviations from ideal spherical micelles; however, the formation of anisotropic or higher-order assemblies should be regarded as a proposed interpretation consistent with AFM observations rather than a directly demonstrated structural pathway.^24,43,44^

At higher levels of organization, the association of micellar assemblies may contribute to the clustered or radially organized features observed by AFM. Nevertheless, these features were obtained from deposited and dried samples and therefore cannot be taken as direct evidence of the native internal organization of micelles in aqueous dispersion. Similarly, DLS supports the presence of a polydisperse hydrated colloidal system in aqueous dispersion, but does not resolve internal architecture or distinguish between specific morphologies such as spherical, elongated, worm-like, or higher-order structures.^45,46^

Therefore, the model presented in Figure 4 should be considered a hypothetical and interpretative scheme based on complementary, indirect characterization data. Definitive confirmation of the internal organization of the micelles would require additional structural and spectroscopic techniques, such as small-angle X-ray scattering, small-angle neutron scattering, nuclear magnetic resonance, fluorescence-based analyses, or cryogenic transmission electron microscopy.

Overall, the combined AFM and self-assembly analyses suggest formulation-dependent differences in the morphology and nanomechanical response of dried/deposited F127-based films. These observations support the proposed interpretative model but should not be considered direct evidence of compact, mechanically resistant, or sterically stabilized micellar nanostructures in aqueous dispersion. Because AFM was performed on deposited samples and SEM on processed dried material, additional solution-state techniques would be required to confirm micellar organization and colloidal stability under aqueous conditions.^20,23^

Additional AFM images of FBC acquired at different scan sizes are provided in Figure S2. These complementary images support the presence of heterogeneous clustered and elongated surface domains in dried/deposited FBC films, while reinforcing that AFM observations should be interpreted as surface-level information rather than direct evidence of hydrated micellar architecture in solution.

### 3.7 Scanning Electron Microscopy Analysis

The morphological features and temporal stability of the formulations were further evaluated by SEM, as shown in Figure 5. The macroscopic appearance of the formulations (Figure 5A) corresponds to FB, containing F127 and the brachydin-enriched fraction; FC, containing F127 and curcumin; and FBC, containing F127 co-loaded with the brachydin-enriched fraction and curcumin, at 72 h after preparation. Figure 5B shows the same formulations after 30 days of storage.

At 72 h after preparation, all formulations appeared macroscopically homogeneous, indicating effective initial dispersion of the hydrophobic bioactive materials within the F127-based system. However, closer visual inspection of the FC formulation already suggested the onset of slight heterogeneity, evidenced by subtle variations in color intensity and the beginning of phase differentiation at the bottom of the vial. This behavior indicates limited macroscopic physical stability at higher nominal formulation concentrations, particularly 250–500 μg mL^–1^, suggesting that curcumin alone may not be fully stabilized within the F127 assemblies over time, likely due to its tendency toward aggregation and recrystallization.

In contrast, the FBC formulation remained clear and visually uniform at 72 h, with no signs of phase separation, turbidity, or sedimentation. This observation suggests improved macroscopic compatibility between the hydrophobic active mixture and the F127-based system. However, because the brachydin component corresponds to an enriched fraction, this behavior should be interpreted as a formulation-level effect associated with co-loading the brachydin-enriched fraction and curcumin, rather than as evidence that individual purified brachydins promote a defined internal core organization.

Over extended visual monitoring for 30 days, formulation-dependent macroscopic differences became more evident. While FC exhibited visible structural changes, the FBC system maintained visual homogeneity, with no evident phase separation, turbidity, or sedimentation under the evaluated conditions. These observations support macroscopic physical integrity of FBC during visual monitoring, but they should not be interpreted as quantitative evidence of long-term physical or colloidal stability.

Importantly, no time-dependent DLS measurements were performed after 30 days of storage. Therefore, possible changes in hydrodynamic diameter, PDI, scattering intensity, or ζ potential during storage cannot be excluded. Similarly, SEM analysis of lyophilized material after storage provides information on the dried matrix morphology, but does not directly represent the hydrated colloidal state of the formulation.

The improved visual behavior of FBC may be associated with the simultaneous incorporation of the brachydin-enriched fraction and curcumin into the F127-based system, which could reduce visible phase separation compared with single-component formulations. Nevertheless, the specific molecular basis of this behavior cannot be definitively assigned from the present data. Therefore, the apparent stability advantage of FBC should be described as improved macroscopic visual integrity under the tested conditions, rather than as conclusive evidence of long-term nanoscale colloidal stability or a fully resolved co-encapsulation mechanism.^21,22^

SEM analysis was performed on the FBC formulation after 30 days of storage, following freezing, lyophilization, drying, and sputter-coating (Figure 5C). The micrograph shows a micrometer-scale porous, irregular, and interconnected dried structure composed of folded and fibrillar-like domains. Therefore, these images should be interpreted strictly as the morphology of the processed dried material and not as a direct representation of hydrated micelles in aqueous dispersion.

The porous and fibrillar-like features observed by SEM may be strongly influenced by sample-processing steps, including freezing, ice-crystal formation, lyophilization, polymer collapse, aggregation, and rearrangement during solvent removal.^39,40^ Consequently, the SEM image cannot be used to validate the native organization of the smaller number-weighted population, anisotropic micelles, worm-like structures, hierarchical micellar networks, or higher-order assemblies in solution.

The fibrillar and entangled features observed by SEM are qualitatively comparable to surface patterns detected by AFM; however, both techniques involve deposited, dried, or processed samples. Thus, they provide information on dried-state morphology and surface organization after solvent removal, whereas DLS provides information on the apparent hydrodynamic behavior of hydrated colloidal populations in aqueous dispersion. These techniques should therefore be considered complementary but not directly interchangeable.

Overall, the SEM results indicate that FBC can form a porous and fibrillar-like dried matrix after freezing and lyophilization, but they do not provide direct evidence of micellar architecture, hierarchical organization, or colloidal networks in solution. Confirmation of any solution-state hierarchical micellar organization would require additional structural techniques, such as cryo-TEM, TEM, SAXS, SANS, or concentration-dependent DLS.

### 3.8 In vitro Cytotoxicity in Mammalian Cells

The cytotoxicity of the formulations was evaluated in GM07492A human lung fibroblasts under both dark and light exposure conditions (Figure 6). In the absence of light, the formulations generally preserved fibroblast viability across the tested concentration range, indicating low basal cytotoxicity under dark conditions.^47^ This result suggests that exposure to the F127-based formulations did not induce pronounced intrinsic toxicity toward mammalian fibroblasts under the evaluated experimental conditions.

Under light exposure, however, fibroblast viability decreased at higher formulation concentrations, indicating that the previous statement that cell viability remained uniformly high under irradiated conditions was overly broad. Therefore, the interpretation was revised to restrict the claim of low cytotoxicity to the concentration range in which fibroblast viability was effectively preserved. At the highest tested concentrations, the reduction in viability should be interpreted as concentration-dependent light-associated cytotoxicity or phototoxic stress in mammalian cells, rather than as negligible phototoxicity. Because no direct ROS assay was performed in fibroblasts, this light-associated reduction in viability should not be mechanistically attributed to ROS generation without additional experimental confirmation.^48,49^

The cytotoxic concentration required to reduce fibroblast viability by 50% (CC_50_) should be reported when the 50% viability threshold is reached within the tested concentration range. When this threshold is not reached, the CC_50_ should be reported as greater than the highest tested concentration, namely CC_50_ > 500 μg mL^–1^. Accordingly, the cytotoxicity profile of each formulation should be interpreted from the complete dose–response behavior under dark and irradiated conditions, rather than from a general statement that viability remained above a fixed threshold across all concentrations.

Importantly, within the same nominal formulation concentration range, FBC did not show evidence of markedly greater cytotoxicity than the corresponding single-loaded systems. This observation suggests that co-loading the brachydin-enriched fraction and curcumin into the F127-based system did not clearly exacerbate mammalian-cell toxicity compared with the individual formulations under the evaluated conditions. However, because the present study did not include formal combination-index analysis and because the brachydin-containing formulations were prepared using an enriched fraction rather than purified individual brachydins, no conclusion regarding synergistic, additive, or antagonistic cytotoxic interactions between defined compounds can be drawn from these data.^50,51^

Taken together, these results support low basal cytotoxicity under dark conditions and an acceptable mammalian-cell viability profile only within the concentration range where viability was maintained. The previous broad claim of high fibroblast viability under both dark and irradiated conditions was therefore moderated. In addition, the safety profile of FBC should be considered preliminary and concentration-dependent, and formal selectivity analysis requires accurate CC_50_ determination or confirmation that CC_50_ was not reached within the tested concentration range.

### 3.9 In vitro Antileishmanial Activity in *L. amazonensis* Promastigotes

The in vitro antileishmanial activity of the formulations was quantified by determining the half-maximal inhibitory concentration (IC_50_) values under both dark and light exposure conditions (Figure 7).^26,52^ As described in the Experimental section, the concentration range used in the biological assays refers to the nominal concentration of each complete formulation dispersion after dilution in culture medium. Therefore, the IC_50_ values reported for FC, FB, and FBC should be interpreted as formulation-based IC_50_ values, not as IC_50_ values normalized to identical isolated concentrations of curcumin, brachydins, or F127.

Under in vitro dark conditions, the formulations exhibited moderate activity, with formulation-based IC_50_ values of 68.46, 45.50, and 72.74 μg mL^–1^ for FC, FB, and FBC, respectively. Among them, FB showed the highest intrinsic activity under the tested nominal formulation exposure, while the co-loaded formulation (FBC) did not demonstrate improved efficacy. These results indicate that, under the present experimental design, co-loading the brachydin-enriched fraction with curcumin did not produce a synergistic antiparasitic effect in the absence of photoactivation. However, because the formulations were compared at equivalent nominal formulation concentrations rather than at equivalent isolated active-compound concentrations, these data should be interpreted as a comparison of complete formulation performance rather than as a direct pharmacological comparison of equal amounts of purified brachydins and curcumin.^25,50^

In contrast, under in vitro light exposure, antileishmanial activity was enhanced mainly in formulations containing the brachydin-enriched fraction. FB showed the strongest light-associated biological response, with IC_50_ decreasing from 45.50 to 18.63 μg mL^–1^, corresponding to a light-associated activity gain of 2.44 (Figure 8). FBC also showed improved activity upon irradiation, with IC_50_ decreasing from 72.74 to 34.33 μg mL^–1^, corresponding to an approximately two-fold gain. In contrast, FC did not improve under light exposure, presenting an IC_50_ of 76.10 μg mL^–1^. Therefore, although curcumin is widely described as a photosensitizer, the curcumin-loaded formulation did not show a measurable photodynamic enhancement under the irradiation and formulation conditions used here.

**Figure 8:**
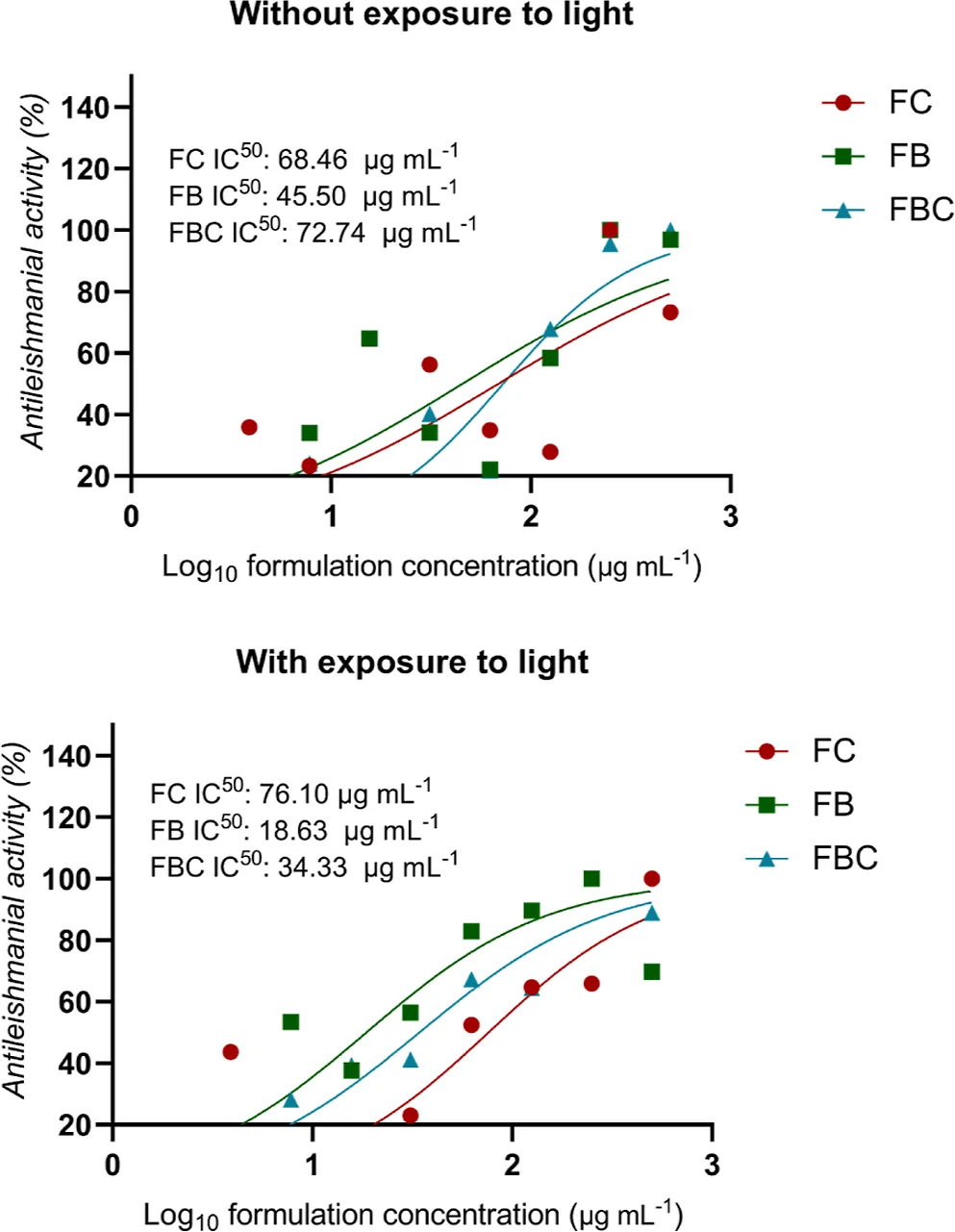
Dose–response curves of FC, FB, and FBC formulations against *L. amazonensis* promastigotes under dark and light conditions. The *x*-axis is presented as log_10_ formulation concentration (μg mL^–1^). IC_50_ values, corresponding to the nominal formulation concentration required to reduce parasite viability by 50%, were determined by nonlinear regression analysis. Results are presented as mean ± standard deviation (SD) from three independent experiments performed in triplicate, with a 95% confidence interval. Formulations containing the brachydin-enriched fraction (FB and FBC) showed enhanced light-associated antileishmanial activity, whereas FC did not show improved activity under irradiation.

These results demonstrate a light-associated antileishmanial response, mainly in formulations containing the brachydin-enriched fraction, but they do not directly demonstrate reactive oxygen species (ROS) generation or confirm a ROS-mediated mechanism. No direct ROS or singlet oxygen assay, such as DCFH-DA, SOSG, DPBF, ESR spin trapping, or an equivalent method, was performed under the same irradiation conditions used in the biological assays. Therefore, ROS involvement should be considered a plausible mechanistic hypothesis based on the light-dependent reduction in parasite viability and on previous reports describing oxidative processes in photodynamic therapy, rather than a mechanism experimentally demonstrated in the present study.^25,26^

Similarly, the enhanced activity observed for FB and FBC under irradiation may be consistent with oxidative stress-related parasite damage, but this interpretation remains indirect. Because no intracellular ROS detection, mitochondrial damage assay, lipid peroxidation analysis, photobleaching analysis, or ultrastructural evaluation was performed, the precise mechanism responsible for the light-associated antileishmanial effect remains to be elucidated. This limitation is particularly relevant because FC did not show improved activity upon irradiation, indicating that the biological response cannot be attributed simply to the presence of curcumin as a photosensitizing compound.

When analyzed together with the cytotoxicity data in mammalian fibroblasts, these results suggest a preliminary and concentration-dependent safety–activity relationship rather than a definitive selectivity profile.^53^ Under dark conditions, the formulations showed low basal cytotoxicity toward fibroblasts, whereas under light exposure, fibroblast viability decreased at higher formulation concentrations. In parallel, antileishmanial activity increased after irradiation, particularly for formulations containing the brachydin-enriched fraction.^14,54^ The vehicle formulation F maintained low cytotoxicity toward fibroblasts and showed no detectable antileishmanial activity, indicating that the biological effects were mainly associated with the incorporated bioactive materials rather than the F127 carrier itself.^18,55^ However, because macrophage cytotoxicity and intracellular amastigote assays were not performed, a formal selectivity index based on a relevant host-cell infection model could not be determined.

Importantly, the co-loaded FBC formulation was not superior to FB in terms of antileishmanial potency. Under light exposure, FB presented the lowest IC50 value (18.63 μg mL^–1^), whereas FBC showed an IC50 of 34.33 μg mL^–1^. Thus, the biological data do not support a synergistic enhancement of antiparasitic activity by the simultaneous incorporation of brachydins and curcumin. In addition, because the present study did not include combination-index analysis or multiple fixed-ratio experiments, no formal conclusion regarding synergism, additivity, or antagonism can be drawn.^50^

The main advantage of FBC should therefore be interpreted more cautiously. Rather than providing the highest antiparasitic potency, FBC represents a compromise formulation that combines light-associated antileishmanial activity, low basal cytotoxicity toward mammalian fibroblasts under dark conditions, high apparent active-material recovery, short-term apparent active-material retention, and macroscopic physical integrity under the evaluated conditions. This distinction is relevant because FB showed greater biological potency, whereas FBC showed a more favorable overall formulation profile, particularly with respect to co-loading feasibility and apparent macroscopic stability.

The lack of superior activity of FBC compared with FB may be related to formulation-dependent factors, including changes in the relative availability of the brachydin-enriched fraction, altered distribution of the active materials within the F127 assemblies, or possible competition between the co-encapsulated compounds for incorporation into hydrophobic domains. However, these possibilities remain hypothetical and require further investigation.

An additional limitation of the biological evaluation is that antileishmanial activity was assessed only against promastigote forms of *L. amazonensis*. Although promastigotes are widely used for initial screening and provide useful preliminary information on antiparasitic activity, intracellular amastigotes within macrophages represent the clinically relevant stage of mammalian infection. Therefore, the present results should be interpreted as preliminary evidence of light-associated antileishmanial activity and cannot be directly extrapolated to intracellular infection models. In addition, because macrophage cytotoxicity and infected-cell assays were not performed, a formal selectivity index based on a relevant host-cell infection model could not be determined. Future studies should evaluate the formulations against intracellular amastigotes in infected macrophages, together with macrophage cytotoxicity and formal selectivity-index determination, to better define therapeutic relevance, selectivity, and intracellular efficacy.^52,56^

Future studies using optimized brachydin-enriched fraction/curcumin ratios, direct mechanistic assays, intracellular amastigote models, macrophage cytotoxicity assays, and formal drug-combination analysis will be necessary to determine whether co-encapsulation can be adjusted to improve antileishmanial potency while preserving the formulation-related advantages observed for FBC.

The comparative safety–efficacy parameters of the formulations under dark and light conditions are summarized in Table 2.

**Table 2:** Comparative safety–efficacy Profile of the Formulations in Mammalian Fibroblasts and *L. amazonensis* Promastigotes Under Dark and Light Conditions.^a^

formulation	fibroblast CC_50_		L. amazonensis IC_50_		light-associated activity gain dark/light	overall profile
	dark	light	dark	light		
	(μg mL^–1^)	(μg mL^–1^)	(μg mL^–1^)	(μg mL^–1^)		
F	>⁡ 500	>⁡ 500	>⁡ 500	>⁡ 500		vehicle control; negligible activity
FC	>⁡ 500	<500	68.46	76.10	0.90	low photoresponse
FB	>⁡ 500	<500	45.50	18.63	2.44	highest antiparasitic potency
FBC	>500	<500	72.74	34.33	2.12	balanced profile; lower potency than FB

a CC_50_ and IC_50_ values refer to the nominal concentration of each complete formulation dispersion after dilution in culture medium

For fibroblast cytotoxicity, CC_50_ values are reported as >⁡500 μg mL^–1^ when the 50% viability threshold was not reached within the tested concentration range. Under light exposure, FC, FB, and FBC showed concentration-dependent reductions in fibroblast viability at higher concentrations; therefore, CC_50_ values are conservatively reported as <500 μg mL^–1^ until exact values are determined by dose–response fitting. Light-associated activity gain was calculated as the ratio between IC_50_ values obtained under dark and light conditions

6 $$light‐associated\;activity\;gain=\frac{{IC}_{50,dark}}{{IC}_{50,light}}$$

Values above 1 indicate enhanced antileishmanial activity upon irradiation, whereas values below 1 indicate absence of light-associated improvement. This parameter describes the biological difference between dark and irradiated conditions and should not be interpreted as direct evidence of ROS generation or a confirmed photodynamic mechanism.^25,26^ Importantly, all IC_50_ values in this table are formulation-based values, calculated from the nominal concentration of each complete formulation dispersion. Therefore, comparisons among FC, FB, and FBC reflect the performance of the complete formulations under equivalent nominal formulation exposure and should not be interpreted as comparisons based on identical isolated concentrations of brachydins, curcumin, or F127. The descriptor assigned to FBC reflects its overall formulation profile under the evaluated conditions and does not indicate superior antileishmanial potency compared with FB.

## 4 Conclusions

In this study, Pluronic F127 micelles co-encapsulating a brachydin-enriched fraction and curcumin (FBC) were developed as a nanostructured platform for light-assisted antileishmanial evaluation. Within the broader literature on PEO–PPO–PEO nanocarriers, the use of Pluronic F127 as a solubilizing platform for poorly water-soluble compounds is well established. Therefore, the main contribution of the present study is not the use of F127 itself, but the associative co-encapsulation of a brachydin-enriched fraction and curcumin in a single F127-based formulation and the preliminary evaluation of its light-associated biological response against *L. amazonensis* promastigotes. The formulation showed high apparent active-material recovery after centrifugation and filtration, short-term apparent active-material retention, and macroscopic physical integrity under the evaluated conditions, supporting the feasibility of incorporating the brachydin-enriched fraction and curcumin into an F127-based system. However, the long-term nanoscale colloidal stability of the formulation was not fully established and should be further investigated by time-dependent DLS monitoring of hydrodynamic diameter, PDI, scattering intensity, and ζ potential during storage.

Biological assays indicated low basal cytotoxicity toward mammalian fibroblasts under dark conditions, whereas light exposure produced concentration-dependent reductions in fibroblast viability at higher formulation concentrations. In parallel, formulations containing the brachydin-enriched fraction exhibited enhanced antileishmanial activity against *L. amazonensis* promastigotes upon light exposure. Importantly, FB showed the highest antiparasitic potency among the tested formulations, indicating that co-encapsulation of the brachydin-enriched fraction with curcumin did not improve antileishmanial efficacy compared with the formulation containing the brachydin-enriched fraction alone. Thus, the advantage of FBC should not be interpreted as superior biological potency, but rather as an overall formulation profile combining light-associated activity in promastigotes, high apparent active-material recovery, short-term apparent active-material retention, and macroscopic physical integrity under the evaluated conditions. Because FB and FBC were prepared using a brachydin-enriched fraction rather than an individually purified brachydin compound, the biological responses should be interpreted as formulation-level effects associated with this fraction.

It should be noted that ROS generation and other photodynamic parameters were not directly measured in this study, and the antileishmanial evaluation was restricted to promastigote forms. Therefore, the involvement of a photodynamic mechanism should be considered a plausible hypothesis based on the light-dependent biological response, rather than a mechanism directly demonstrated by the present data, and the biological relevance of these findings must still be confirmed in intracellular amastigote models. Future studies should include direct ROS detection, singlet oxygen assays, photobleaching analysis, mechanistic evaluation of parasite damage, infected macrophage assays, and formal combination analyses using optimized brachydin/curcumin ratios to determine whether co-encapsulation can improve biological potency while preserving the formulation-related advantages observed for FBC.

Overall, these findings support FBC as a preliminary associative F127-based formulation for light-assisted antileishmanial investigation, rather than as a confirmed selective photodynamic therapy. It should be emphasized that the self-assembly pathway proposed in this study represents a plausible structural model based on indirect and complementary characterization data, rather than a definitive determination of the internal micellar architecture. Future investigations using advanced structural and spectroscopic techniques will be necessary to confirm the organization of the FBC micellar system in aqueous dispersion. In addition, direct ROS measurements, intracellular amastigote assays, formal selectivity analysis, time-dependent colloidal stability studies, and in vivo evaluation will be required to define the mechanism of action and therapeutic relevance of the formulation.

## Supplementary Material


