# Supplementary material for: Pluronic F127 Micelles Co-encapsulating a Brachydin-Enriched Fraction and Curcumin: A Self-assembled Platform for Light-assisted Antileishmanial Activity
Source: ACS Omega. 2026 Oct 1;11(40):61032–51. doi: 10.1021/acsomega.6c05278 (PMC13670344; doi:10.1021/acsomega.6c05278)
Supplement: Supplementary file 1 [file ao6c05278_si_001.pdf]

Supporting Information

Pluronic F127 Micelles Co-Encapsulating a Brachydin-Enriched Fraction and Curcumin: A Self-Assembled Platform for Light-Assisted Antileishmanial Activity

Ingrid Maria Ribeiro de Lima[1], Aline Santana Figueredo[2], Lucilene Amorim Silva[2], Luciana Magalhães Rebelo Alencar[3], Cláudia Quintino da Rocha[1], Renato Sonchini Gonçalves[4]

[1] Laboratory of Chemistry of Natural Products, Department of Chemistry, Federal University of Maranhão, São Luís 65080-805, Brazil

[2] Laboratory of Immunophysiology, Center for Biological and Health Sciences, Federal University of Maranhão, São Luís 65080-805, Brazil

[3] Laboratory of Biophysics and Nanosystems, Department of Physics, Federal University of Maranhão, São Luís 65080-805, Brazil

[4] Department of Engineering and Exact Sciences, Palotina Sector, Federal University of Paraná (UFPR), Palotina 85950-000, PR, Brazil

(A)

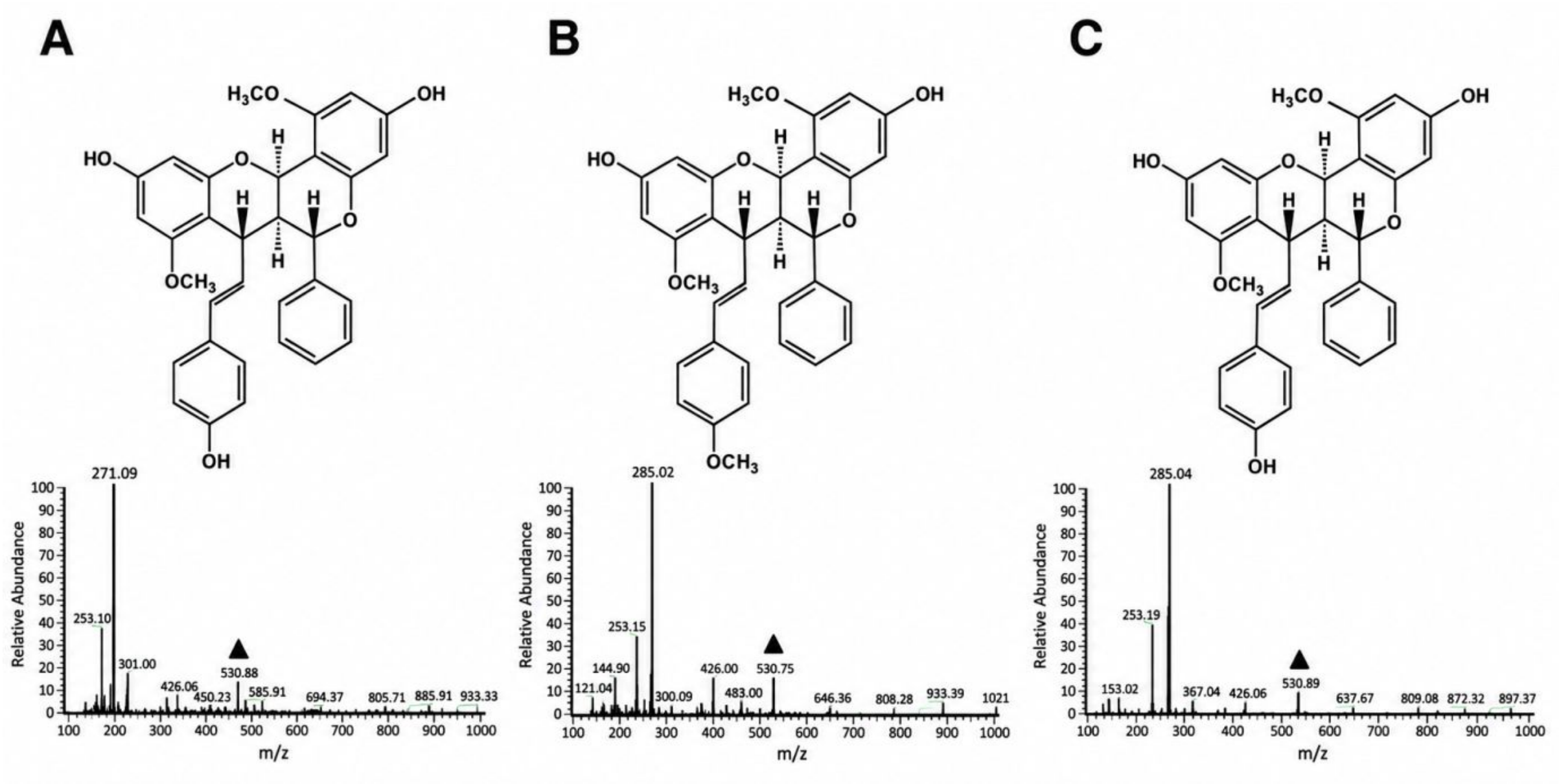

(B)

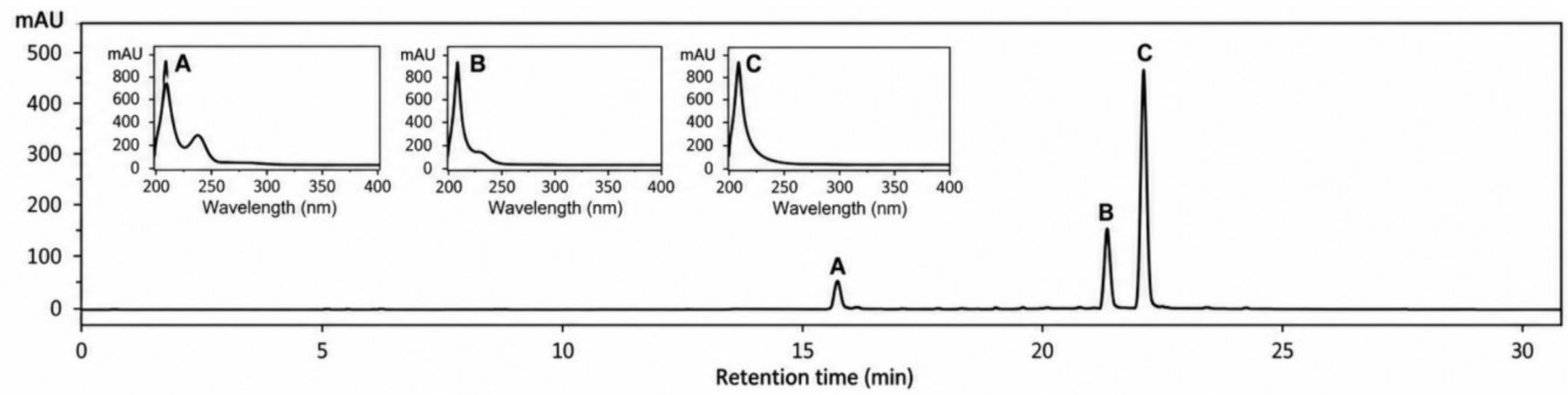

Figure S1. Representative chromatographic and spectrometric profile of the brachydin-enriched dichloromethane fraction obtained from the roots of *Fridericia platyphylla* (formerly *Arrabidaea brachypoda*). **(A)** Chemical structures and corresponding mass spectra of the three major dimeric flavonoids previously assigned as brachydin A, brachydin B, and brachydin C. **(B)** HPLC-PDA chromatogram of the brachydin-enriched fraction recorded at 254 nm, showing the chromatographic peaks assigned to brachydins A, B, and C. The corresponding UV absorption spectra are shown as insets.

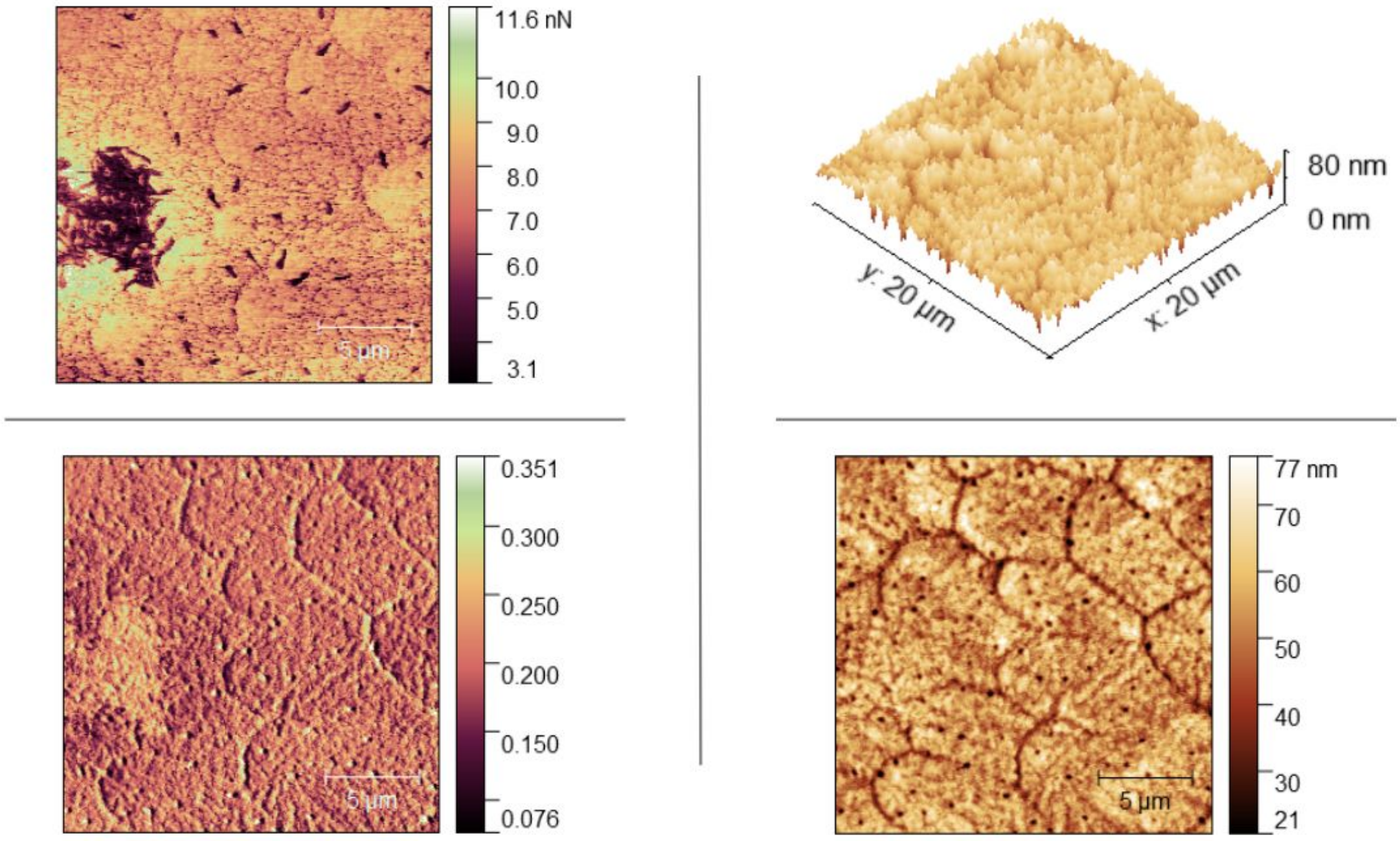
11.6 nN
10.0
9.0
8.0
7.0
6.0
5.0
3.1
5 µm
80 nm
0 nm
y: 20 µm
x: 20 µm
0.351
0.300
0.250
0.200
0.150
0.076
5 µm
77 nm
70
60
50
40
30
21
5 µm

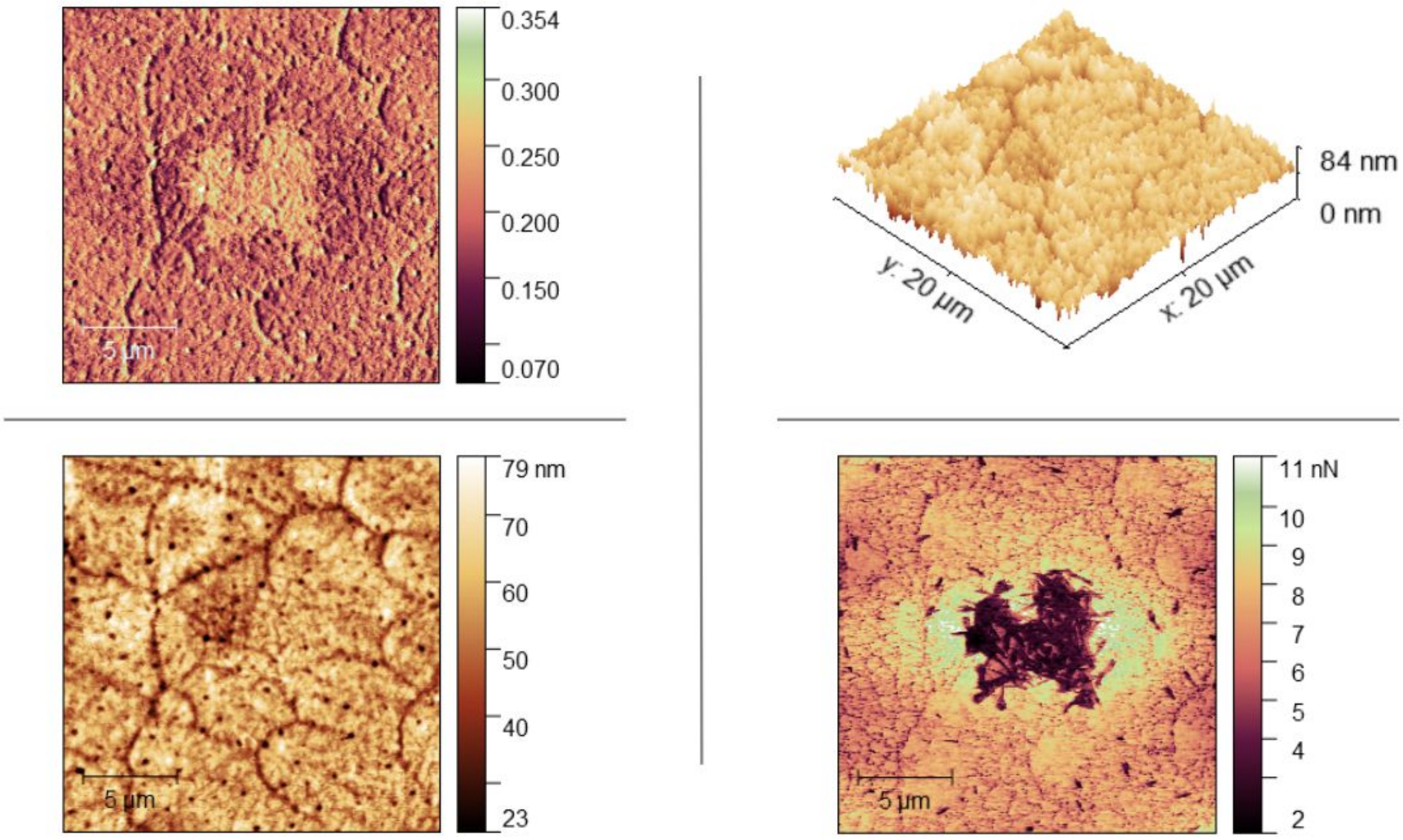
0.354
0.300
0.250
0.200
0.150
0.070
5 µm
84 nm
0 nm
y: 20 µm
x: 20 µm
79 nm
70
60
50
40
23
5 µm
11 nN
10
9
8
7
6
5
4
2
5 µm

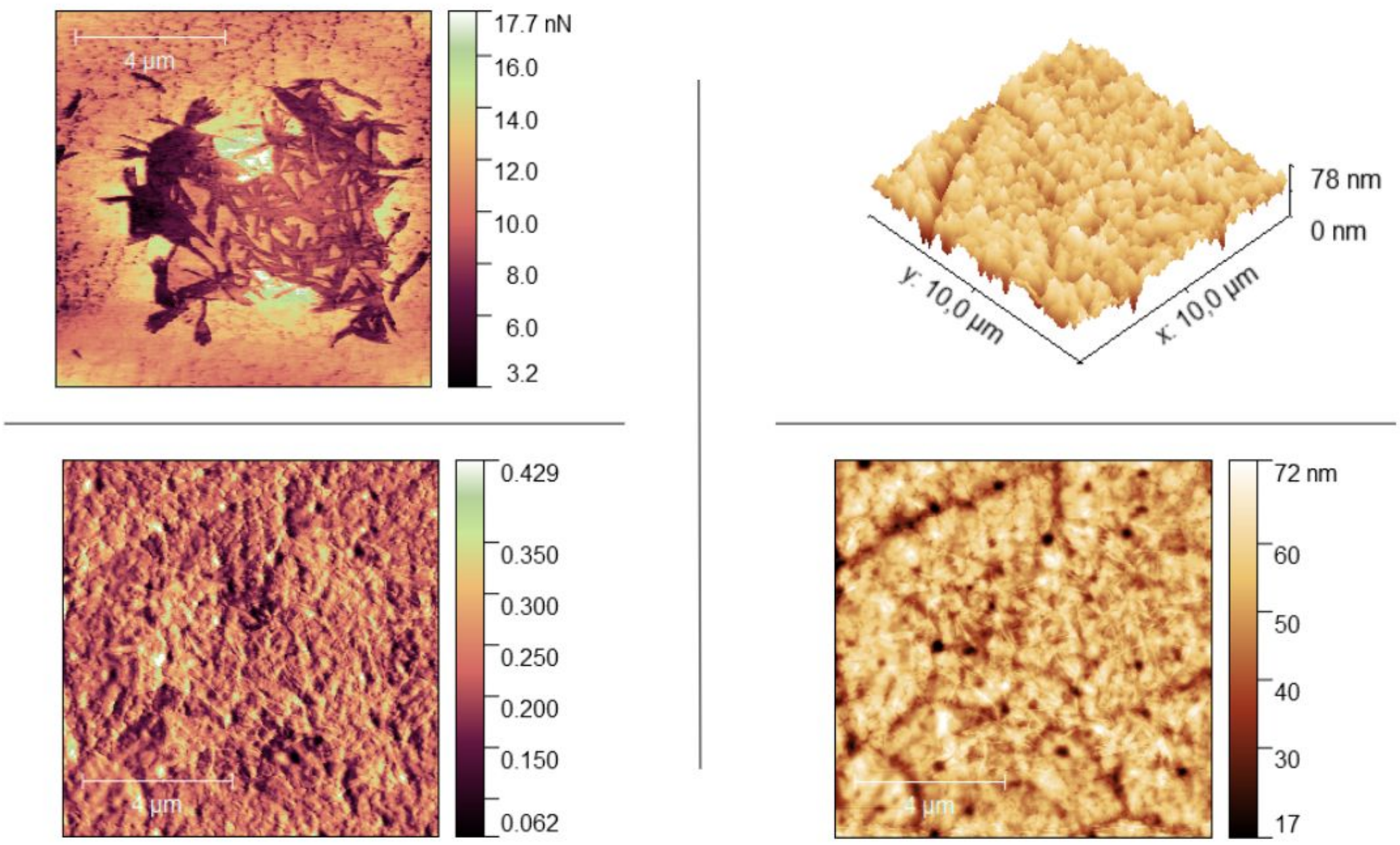
17.7 nN
16.0
14.0
12.0
10.0
8.0
6.0
3.2
4 µm
78 nm
0 nm
y: 10,0 µm
x: 10,0 µm
0.429
0.350
0.300
0.250
0.200
0.150
0.062
4 µm
72 nm
60
50
40
30
17
4 µm

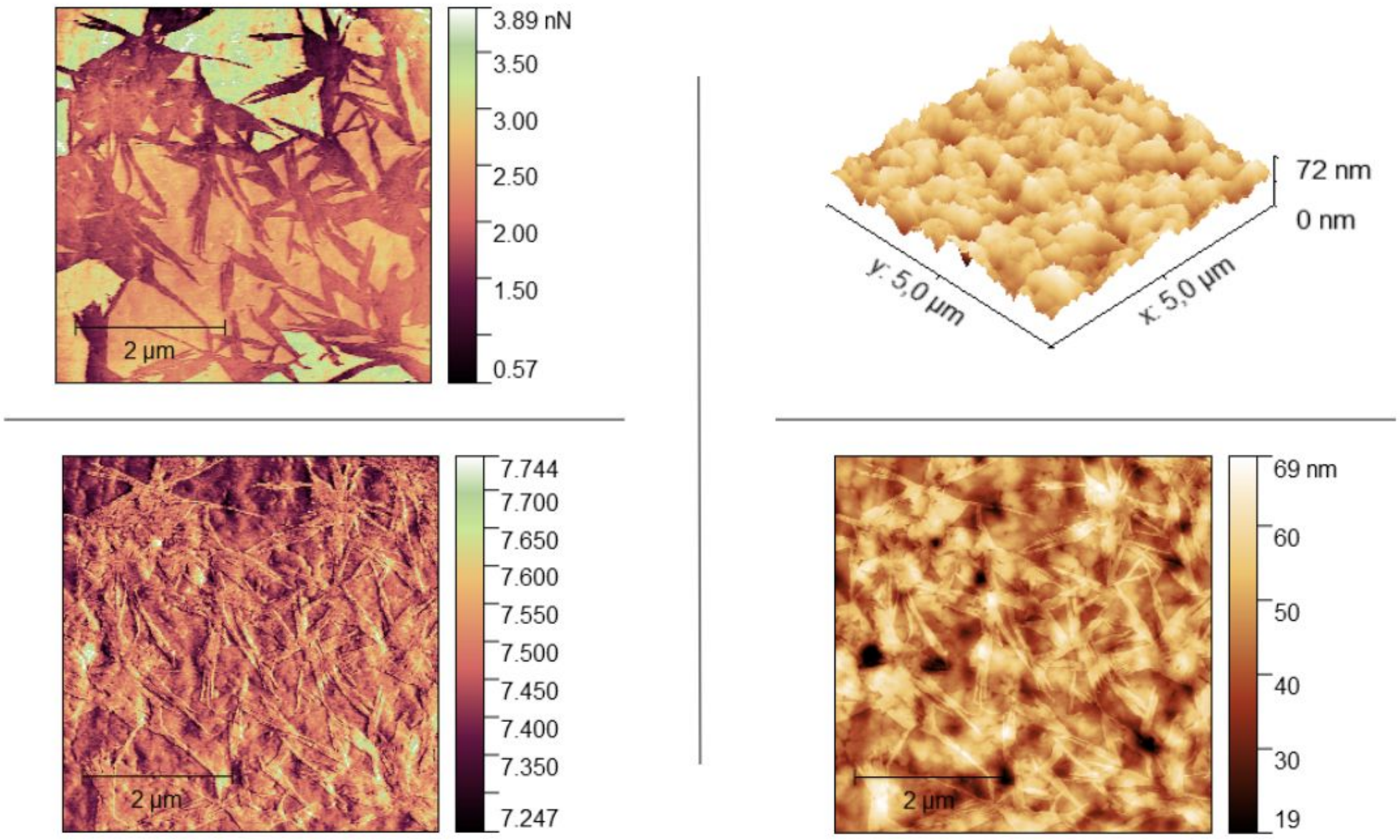
3.89 nN
3.50
3.00
2.50
2.00
1.50
0.57
2 µm
72 nm
0 nm
y: 5,0 µm
x: 5,0 µm
7.744
7.700
7.650
7.600
7.550
7.500
7.450
7.400
7.350
7.247
2 µm
69 nm
60
50
40
30
19
2 µm

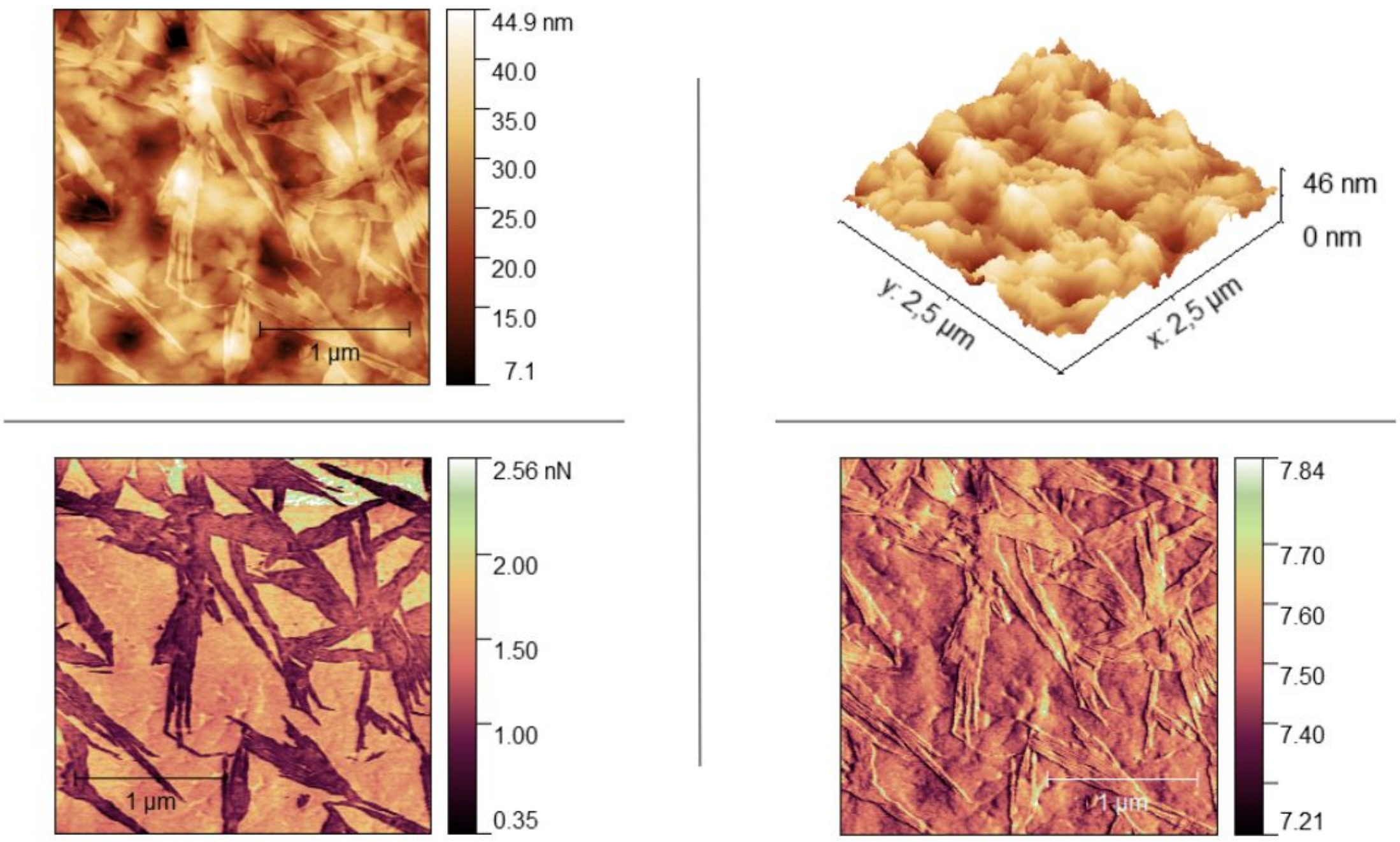
44.9 nm
40.0
35.0
30.0
25.0
20.0
15.0
7.1
1 µm
46 nm
0 nm
y: 2,5 µm
x: 2,5 µm
2.56 nN
2.00
1.50
1.00
0.35
1 µm
7.84
7.70
7.60
7.50
7.40
7.21
1 µm

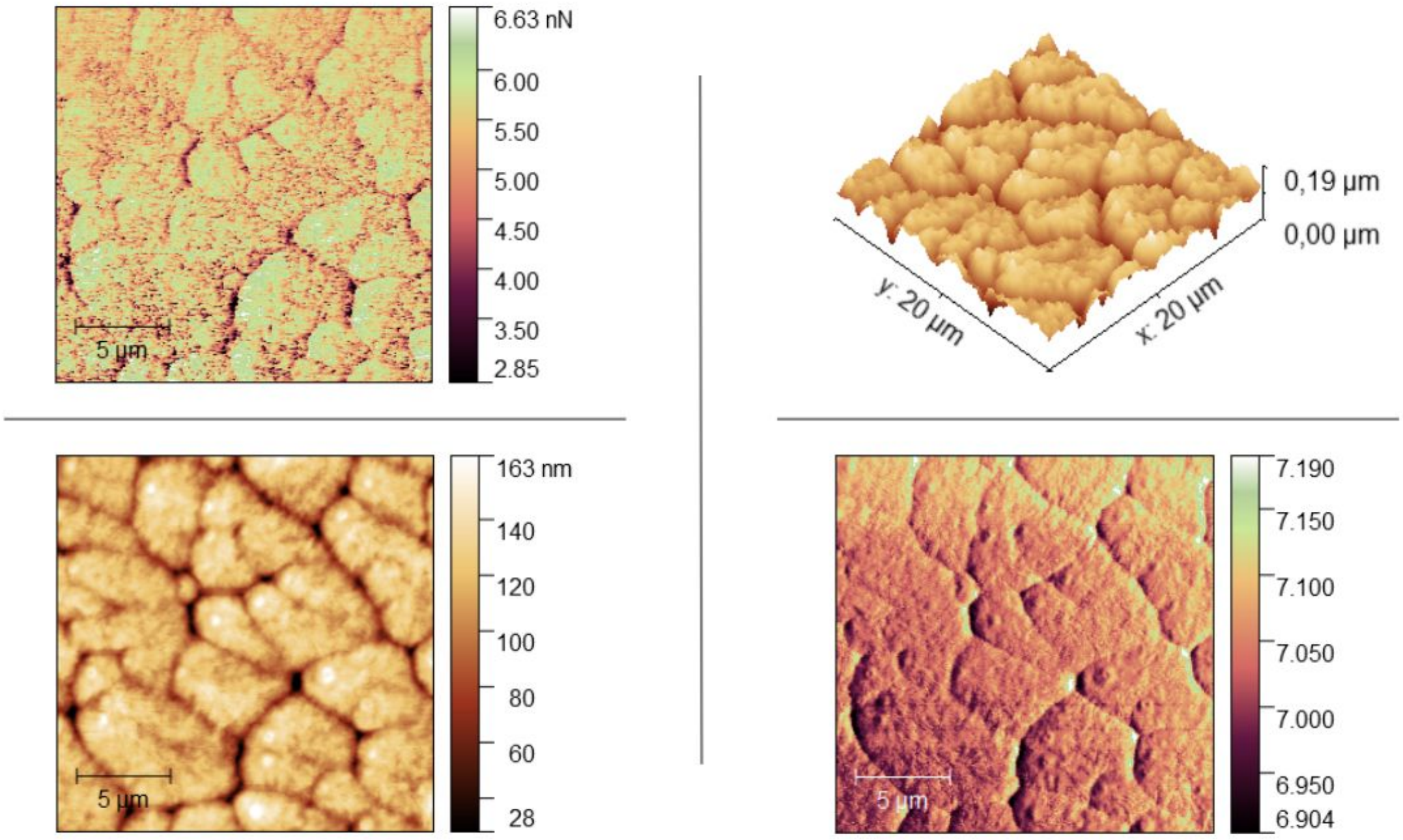

6.63 nN
6.00
5.50
5.00
4.50
4.00
3.50
2.85
5 µm
0,19 µm
0,00 µm
y: 20 µm
x: 20 µm
163 nm
140
120
100
80
60
28
5 µm
7.190
7.150
7.100
7.050
7.000
6.950
6.904
5 µm

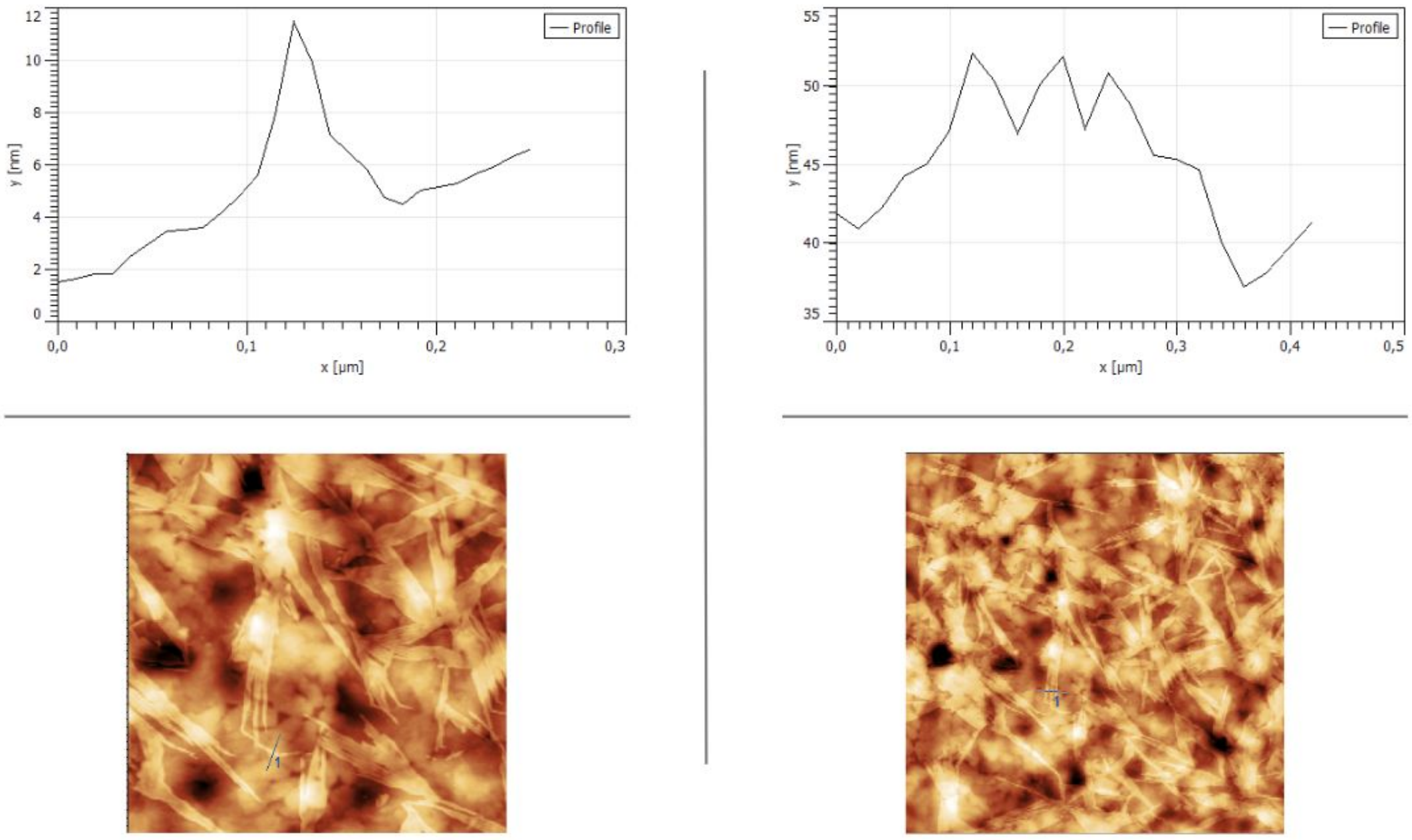

Profile
y [nm]
x [µm]
0,0
0,1
0,2
0,3
Profile
y [nm]
x [µm]
0,0
0,1
0,2
0,3
0,4
0,5

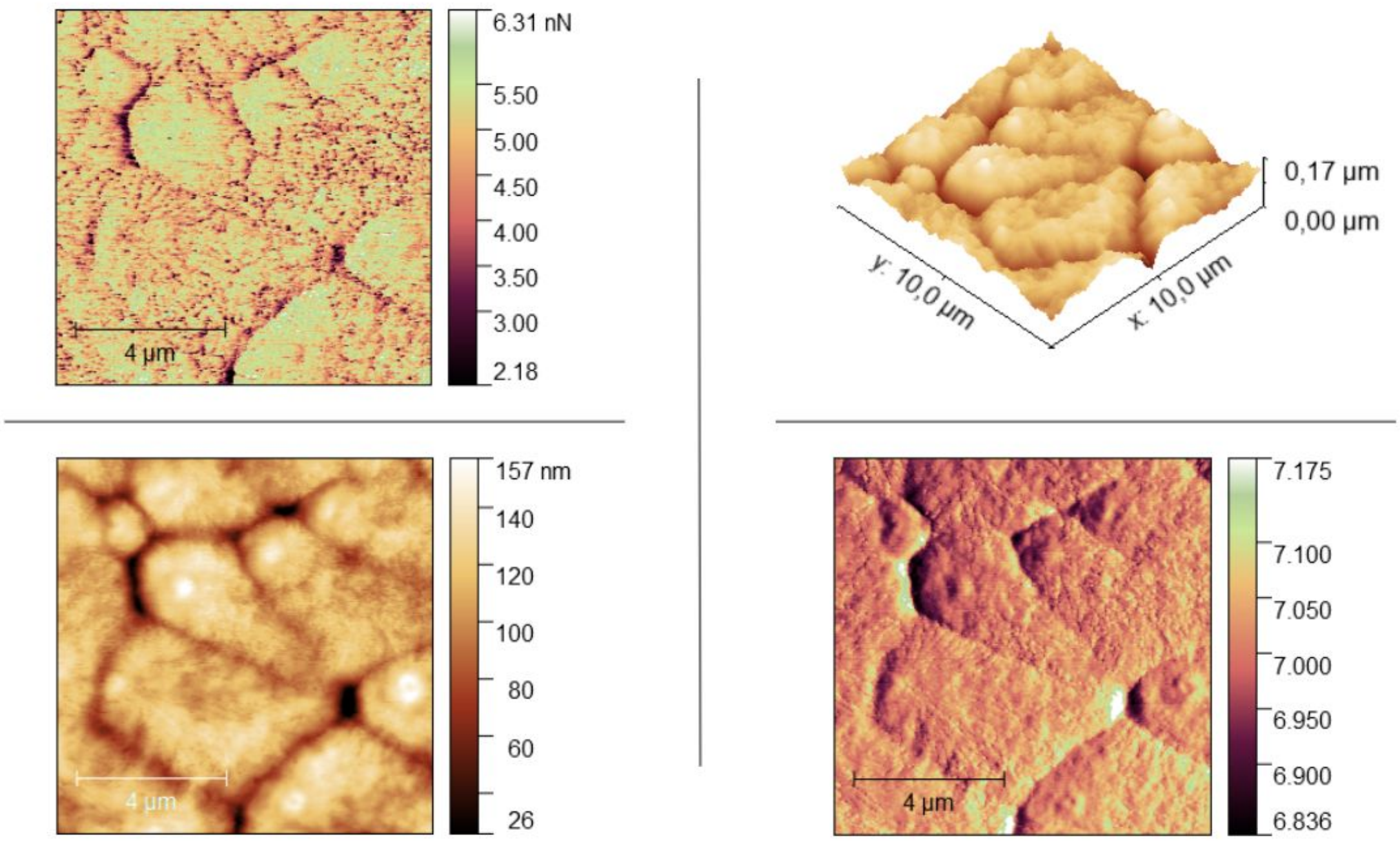
6.31 nN
5.50
5.00
4.50
4.00
3.50
3.00
2.18
4 µm
0,17 µm
0,00 µm
y: 10,0 µm
x: 10,0 µm
157 nm
140
120
100
80
60
26
4 µm
7.175
7.100
7.050
7.000
6.950
6.900
6.836
4 µm

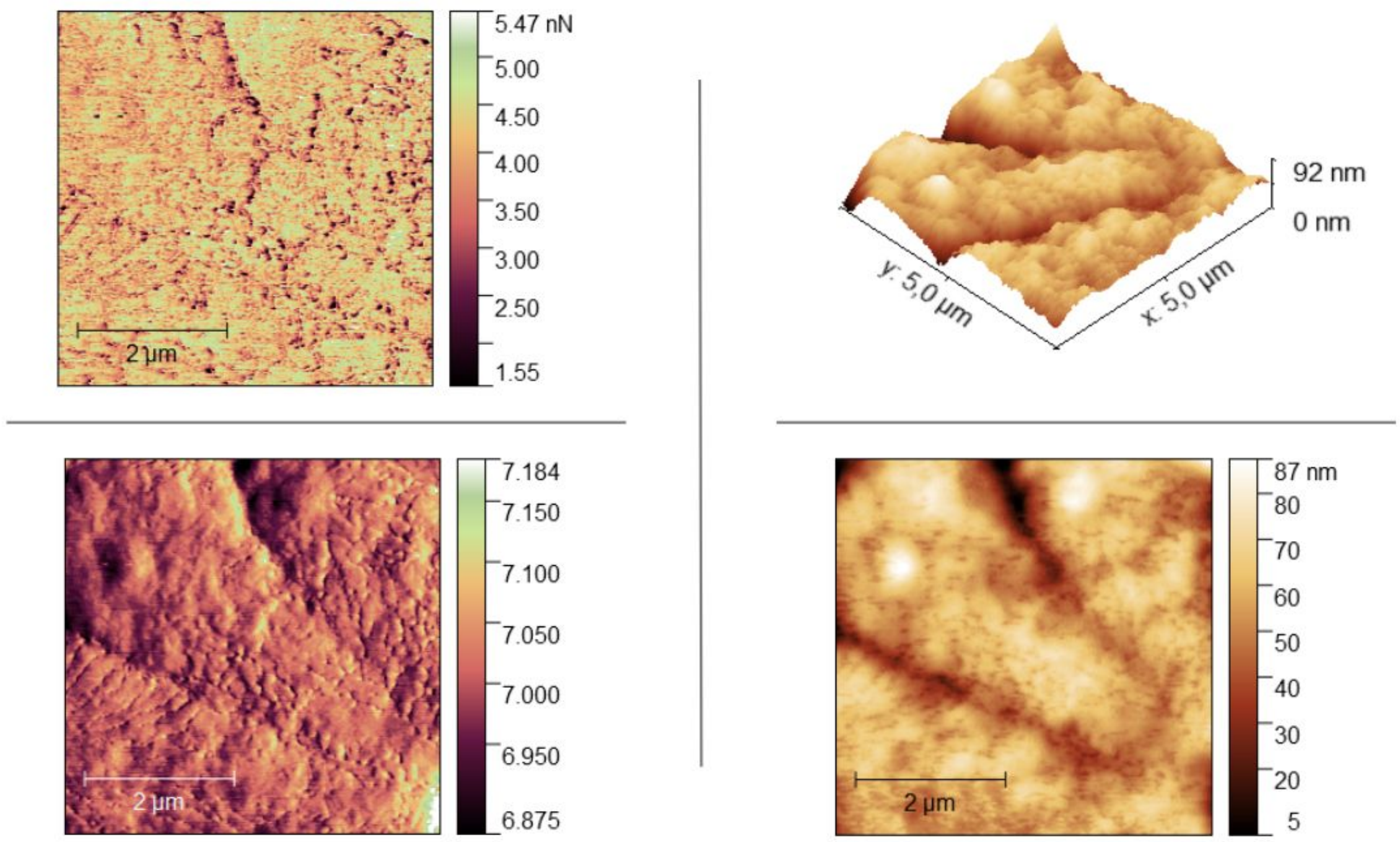
5.47 nN
5.00
4.50
4.00
3.50
3.00
2.50
1.55
2 µm
92 nm
0 nm
y: 5,0 µm
x: 5,0 µm
7.184
7.150
7.100
7.050
7.000
6.950
6.875
2 µm
87 nm
80
70
60
50
40
30
20
5
2 µm

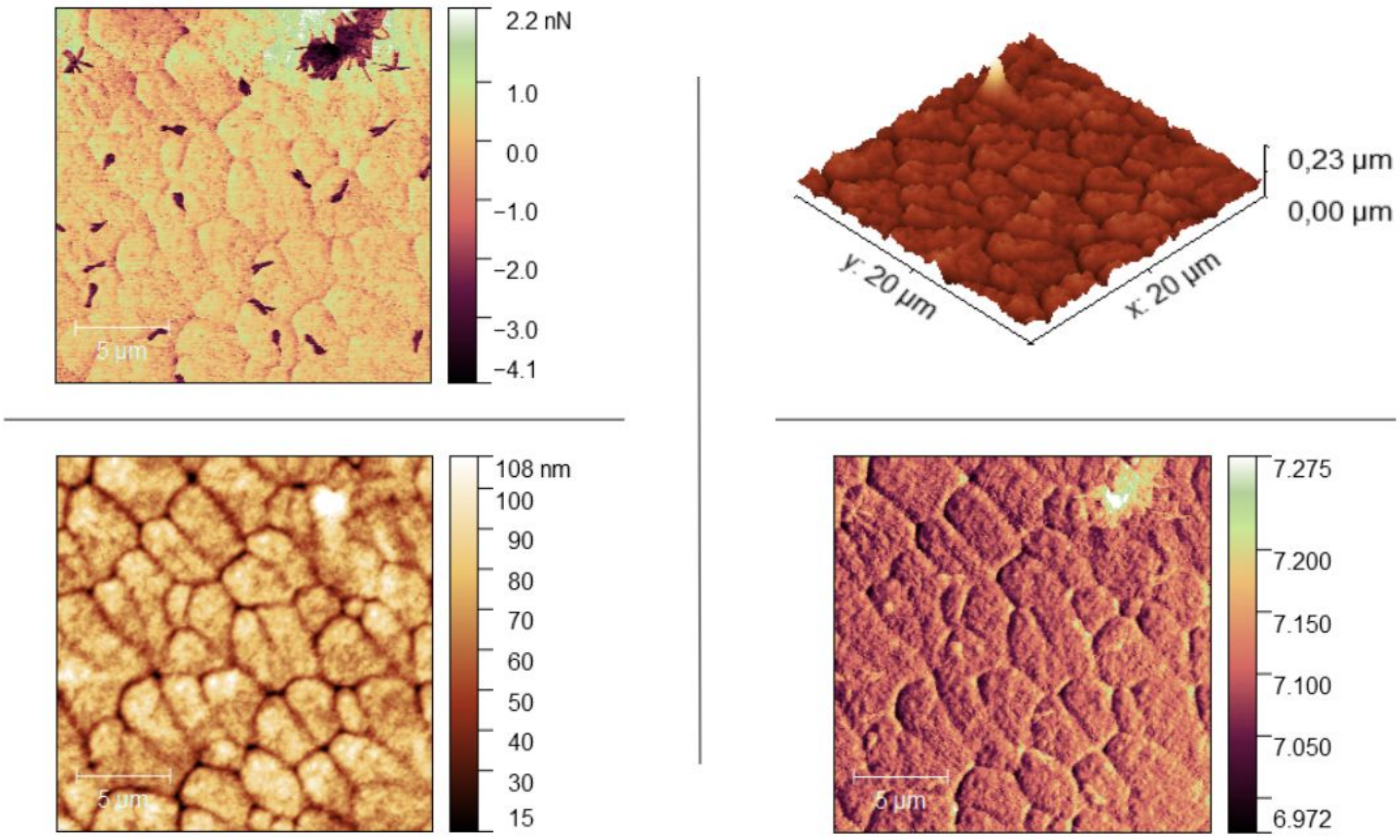
2.2 nN
1.0
0.0
−1.0
−2.0
−3.0
−4.1
5 µm
0,23 µm
0,00 µm
y: 20 µm
x: 20 µm
108 nm
100
90
80
70
60
50
40
30
15
5 µm
7.275
7.200
7.150
7.100
7.050
6.972
5 µm

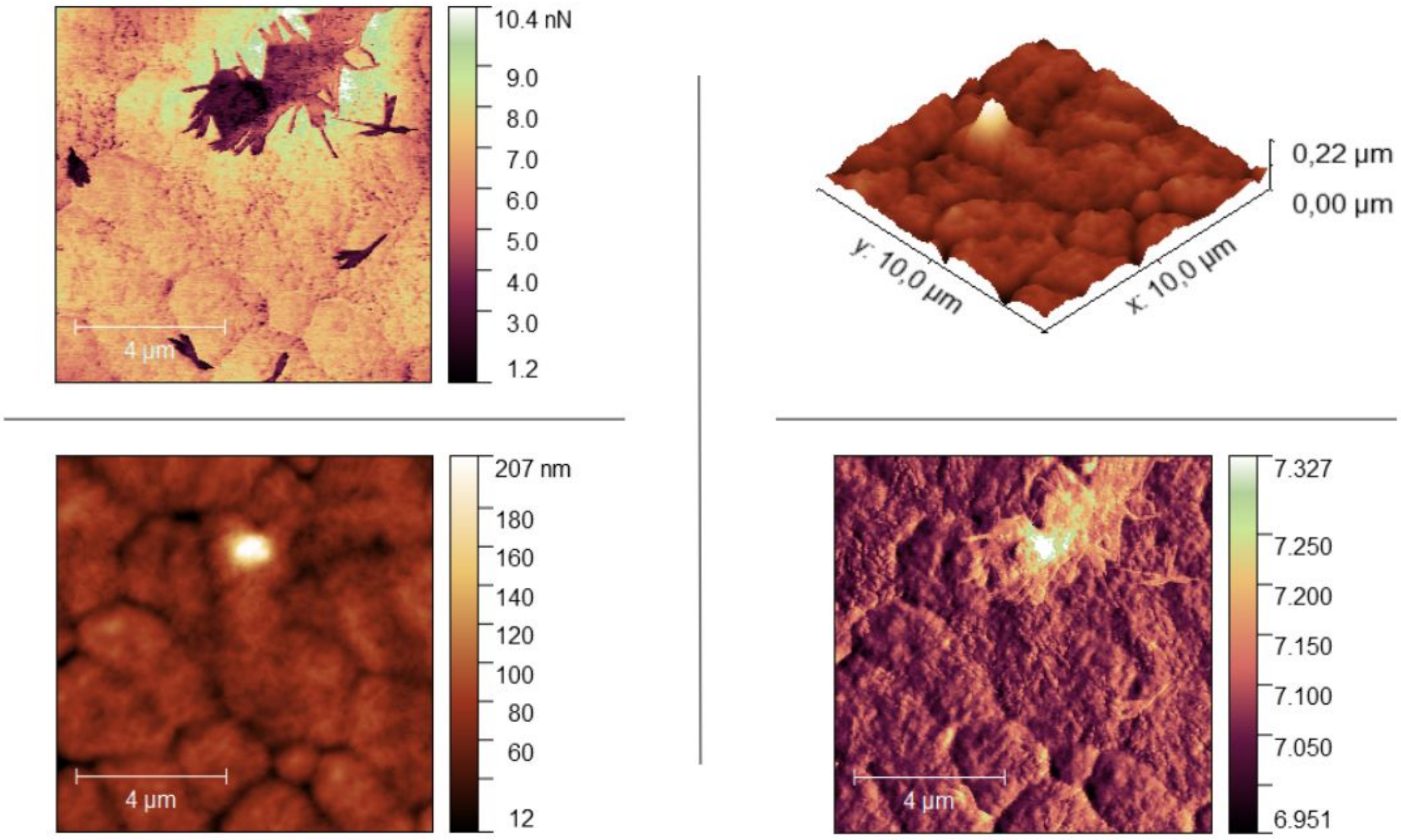
10.4 nN
9.0
8.0
7.0
6.0
5.0
4.0
3.0
1.2
4 µm
0,22 µm
0,00 µm
y: 10,0 µm
x: 10,0 µm
207 nm
180
160
140
120
100
80
60
12
4 µm
7.327
7.250
7.200
7.150
7.100
7.050
6.951
4 µm

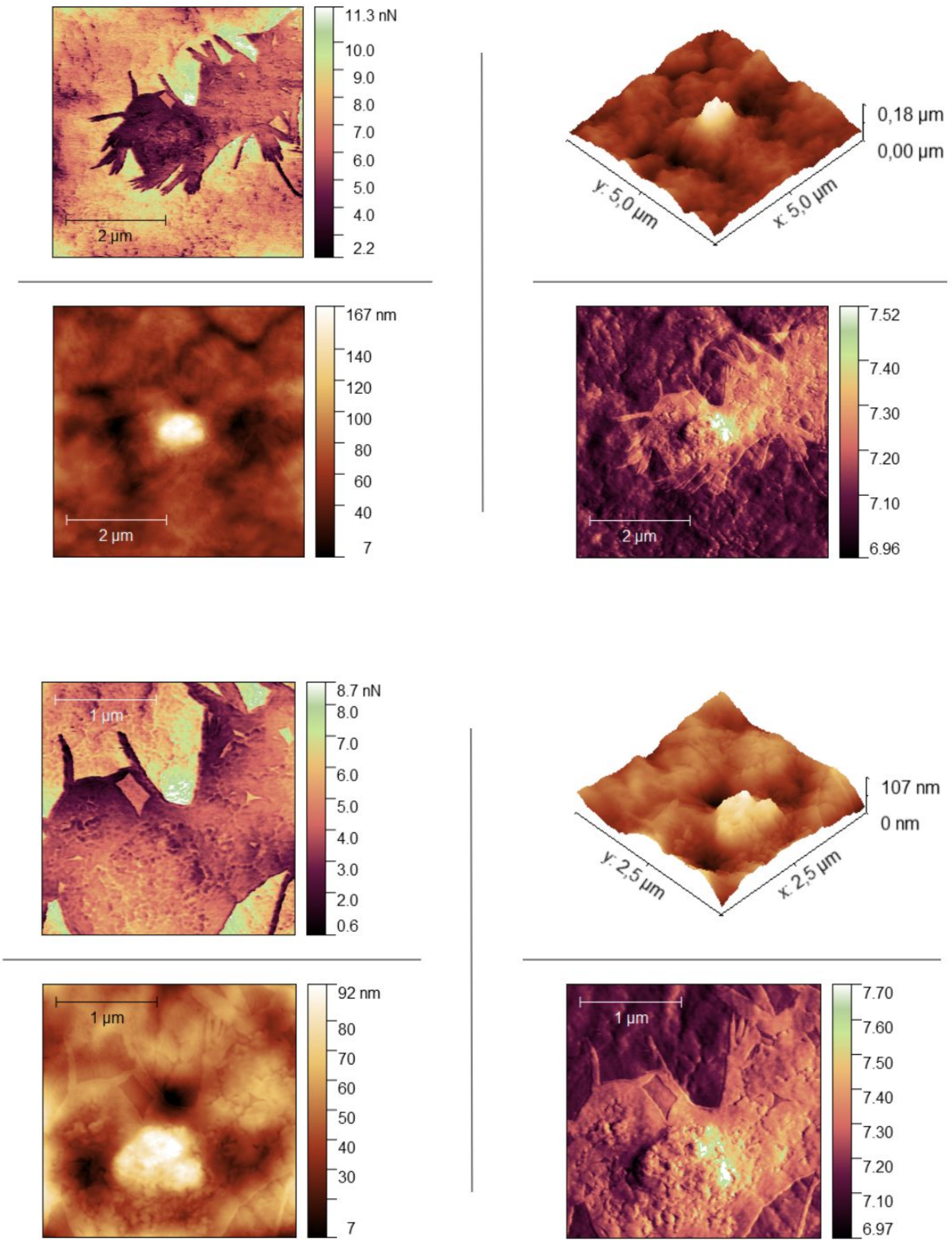

Figure S2. Complementary AFM characterization of the FBC formulation after deposition on mica. Representative height/topography maps, three-dimensional surface renderings, apparent nanomechanical maps, adhesion-force maps, and section profiles are shown at different scan sizes. The images reveal formulation-dependent surface heterogeneity, including clustered, elongated, and locally coalesced domains in the dried/deposited F127-based film. These complementary AFM data support the qualitative interpretation that FBC forms organized

surface assemblies after deposition and drying. However, because the measurements were performed on deposited samples, these images should not be interpreted as direct evidence of hydrated micelles, micellar fusion, or native micellar architecture in aqueous dispersion.

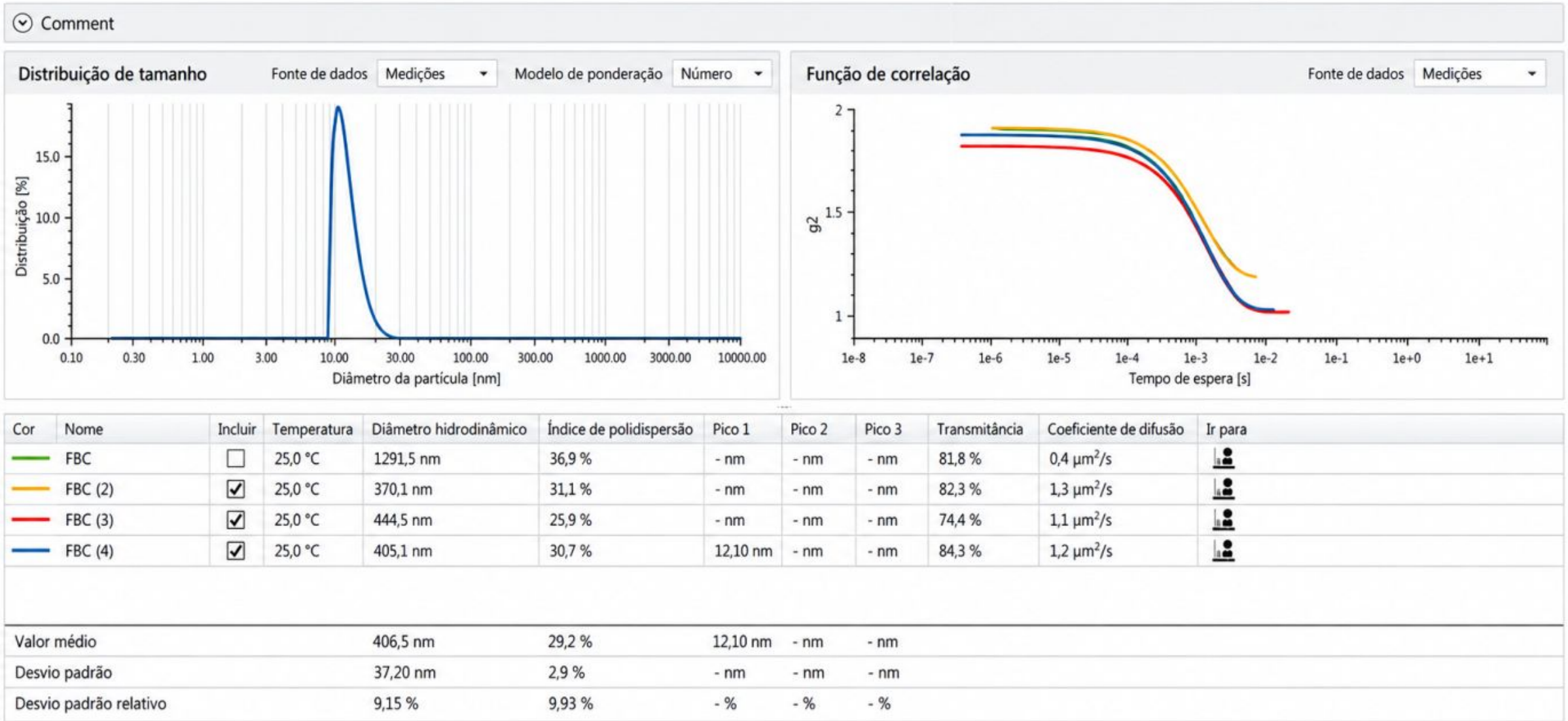

Cor	Nome	Incluir	Temperatura	Diâmetro hidrodinâmico	Índice de polidispersão	Pico 1	Pico 2	Pico 3	Transmitância	Coeficiente de difusão	Ir para
	FBC	☐	25,0 °C	1291,5 nm	36,9 %	- nm	- nm	- nm	81,8 %	0,4 µm²/s	
	FBC (2)	☑	25,0 °C	370,1 nm	31,1 %	- nm	- nm	- nm	82,3 %	1,3 µm²/s	
	FBC (3)	☑	25,0 °C	444,5 nm	25,9 %	- nm	- nm	- nm	74,4 %	1,1 µm²/s	
	FBC (4)	☑	25,0 °C	405,1 nm	30,7 %	12,10 nm	- nm	- nm	84,3 %	1,2 µm²/s	
Valor médio				406,5 nm	29,2 %	12,10 nm	- nm	- nm			
Desvio padrão				37,20 nm	2,9 %	- nm	- nm	- nm			
Desvio padrão relativo				9,15 %	9,93 %	- %	- %	- %			

Figure S3. DLS report of the FBC formulation. **On the left**, the number-weighted size-distribution graph is shown, highlighting the main population centered at approximately 12.1 nm. **On the right**, the correlation function obtained for the included measurements is presented. **In the lower panel**, the individual measurements used to calculate the apparent mean hydrodynamic diameter and polydispersity index (PDI), together with the corresponding summary statistics, are shown. The included measurements yielded an apparent mean D_h of 406.5 ± 37.2 nm, with PDI ≈ 0.292.
